# Design of novel potent selective survivin inhibitors using 2D-QSAR modeling, molecular docking, molecular dynamics, and ADMET properties of new MX-106 hydroxyquinoline scaffold derivatives

**DOI:** 10.1016/j.heliyon.2024.e38383

**Published:** 2024-09-26

**Authors:** Mourad Aloui, Mohamed El fadili, Somdutt Mujwar, Sara Er-rahmani, Hatem A. Abuelizz, Mohammed Er-rajy, Sara Zarougui, Menana Elhallaoui

**Affiliations:** a LIMAS Laboratory, Faculty of Sciences Dhar El Mahraz, Sidi Mohamed Ben Abdellah University, Fez, Morocco; b Chitkara College of Pharmacy, Chitkara University, Rajpura, 140401, Punjab, India; c Dipartimento di Chimica, Università di Torino, 10125, Torino, Italy; d Department of Pharmaceutical Chemistry, College of Pharmacy, King Saud University, PO Box 2457, Riyadh, 11451, Saudi Arabia

**Keywords:** Breast cancer, QSAR, ADMET properties, Tumor cells, Molecular dynamics, Selective survivin inhibitors, Molecular docking, MX-106 hydroxyquinoline scaffold

## Abstract

Given the critical role of survivin (BIRC5) in tumor cell regulation, developing novel inhibitors represents a promising approach for cancer therapy. This study details the design of innovative survivin inhibitors based on the hydroxyquinoline scaffold of our previously reported lead compound, MX-106. Our study identified nine compounds whose inhibitory activity is expected to be superior to that of the most active molecule in the series. These compounds demonstrated potent suppression of MDA-MB-435 breast cancer cell proliferation in vitro and exhibited enhanced metabolic stability compared to the series' most active member. To evaluate these derivatives as potential survivin inhibitors, we employed a multi-faceted approach combining 2D-QSAR methods, molecular docking, molecular dynamics, and ADMET property assessment. Our molecular modeling studies led to the design of nine novel compounds (Pred1-Pred9) predicted to exhibit potent survivin inhibitory activity based on MLR models. To assess their suitability as drug candidates, we recommend a thorough evaluation of their ADMET properties. These compounds hold promise as innovative anticancer agents targeting survivin, similar to the established MX-106.

## 1 Introduction

The molecular modeling of QSAR types offers a powerful approach to studying survivin inhibitors of breast cancer in medicinal chemistry [1,2]. By linking molecular structure with biological activity, QSAR paves the way for designing more effective and targeted drugs [3]. Overexpressed in many breast cancers, the anti-apoptotic protein survivin presents a promising therapeutic target [4,5]. This protein is selectively disrupted by survivin inhibitors, which cause cancer cells to undergo programmed cell death, or apoptosis [[6], [7], [8]]. The QSAR methods that were used for this study enabled a quantitative analysis of the complex link between inhibitor structures and their ability to modulate survivin activity [9]. This quantitative understanding helps in designing more potent and effective survivin inhibitors [10]. QSAR uses molecular descriptors such as lipophilicity and geometry to build predictive models [11]. These models predict the activity of survivin inhibitors based on their structures and guide the design of new, more potent compounds [12]. A molecular docking analysis was performed to predict the optimal binding mode of a ligand, identify active sites, and understand the interactions between a highly active ligand and its target protein [13]. To gain further insight, we performed molecular dynamics (MD) simulations to investigate the details and stability of these interactions. A 100 ns MD simulation was conducted to evaluate the stability of the (ligand-protein) complex with both newly designed and highly active molecules [14]. Additionally, in silico absorption, distribution, metabolism, excretion, and toxicity (ADMET) studies have been carried out to explore the potential of the new compound as an anticancer drug and to predict its pharmacokinetic and toxicological profiles. This has empowered the development of more targeted and personalized drugs for treating breast cancer.

Survivin's pivotal role in regulating apoptosis, mitosis, and cell cycle progression in cancer cells has made it a prime therapeutic target [5]. Numerous compounds have been developed to inhibit survivin expression, with YM155 being a well-known example [15,16]. However, YM155's effectiveness is limited by its interaction with the drug efflux pump P-glycoprotein (P-gp) [17]. Building on this knowledge, structure-activity relationship (SAR) studies of UC-112 led to the development of MX-106, a derivative with enhanced antitumor activity against melanoma [18]. Mechanistic investigations revealed that MX-106 selectively suppresses survivin expression, inducing apoptosis in cancer cells. Notably, the 8-hydroxyquinoline moiety of MX-106 is crucial for its antitumor effects.

This introduction seeks to highlight the use of QSAR methods in the study of survivin inhibitors for the treatment of breast cancer by emphasizing their central role in the search for innovative pharmaceutical compounds. To better understand the primary structural requirements and analyze the important interactions between the candidate ligand and the targeted receptor, molecular docking studies were also conducted for these compounds towards the survivin receptor coded by 3UIH.pdb. Assess the physiological stability of the ligand-receptor complex, 100 ns molecular dynamics (MD) simulations were conducted. Each designed molecule was also examined using conventional computational pharmacokinetic parameters (ADMET) to assess its pharmacological potential. These approaches offered a crucial perspective for accelerating the drug discovery process by contributing to an in-depth understanding of structure-activity relationships (SAR) and paving the way for more effective and better-tolerated therapies.

## 2 Materials and methods

### 2.1 Experimental dataset

Experimental data on 31 new selective survivin inhibitors derived from the MX-106 hydroxyquinoline scaffold are presented in Table 1.

**Table 1:**
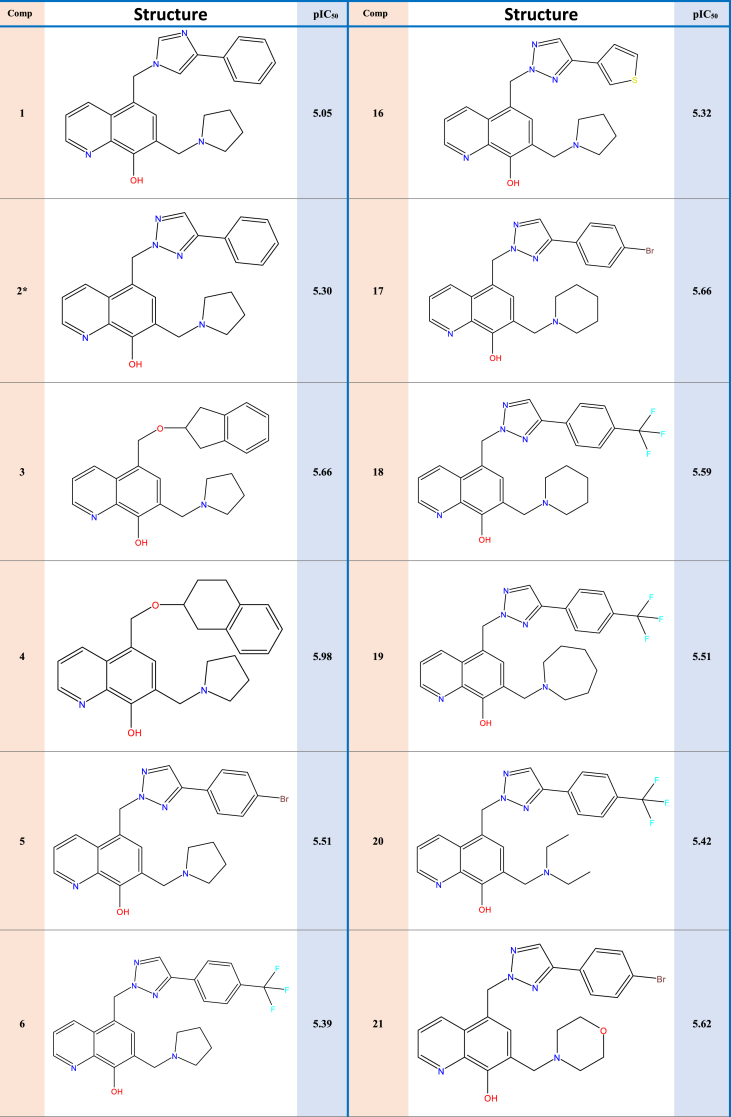

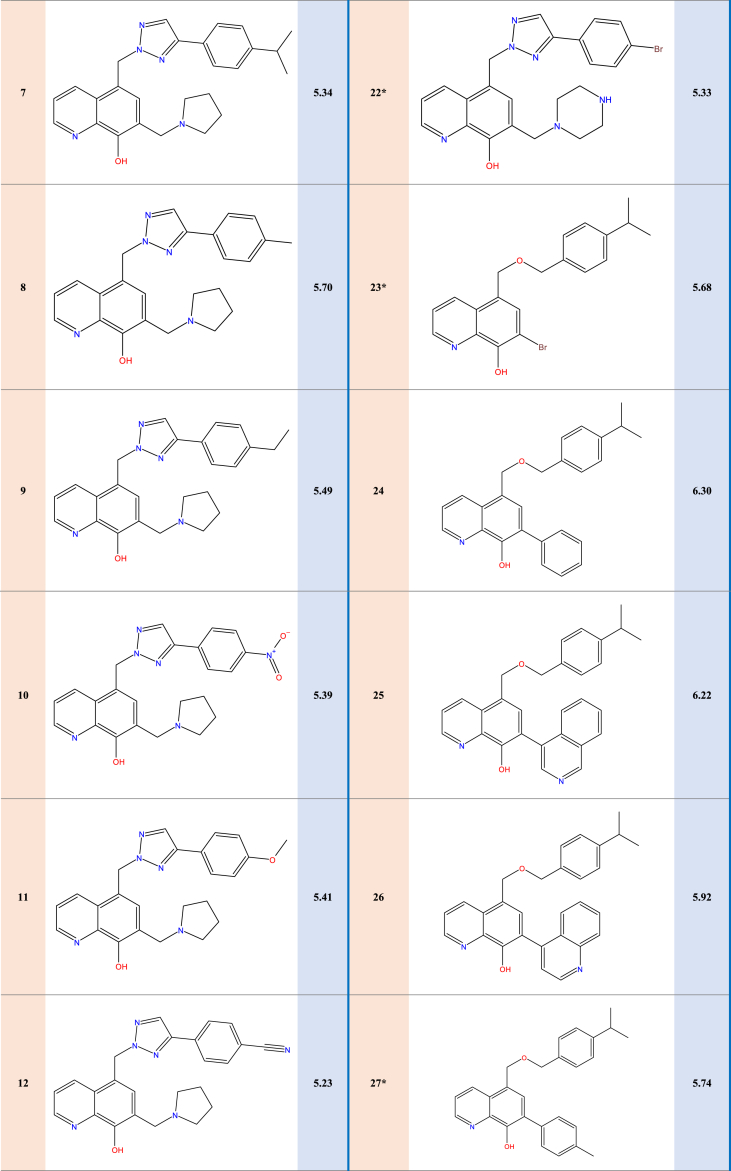

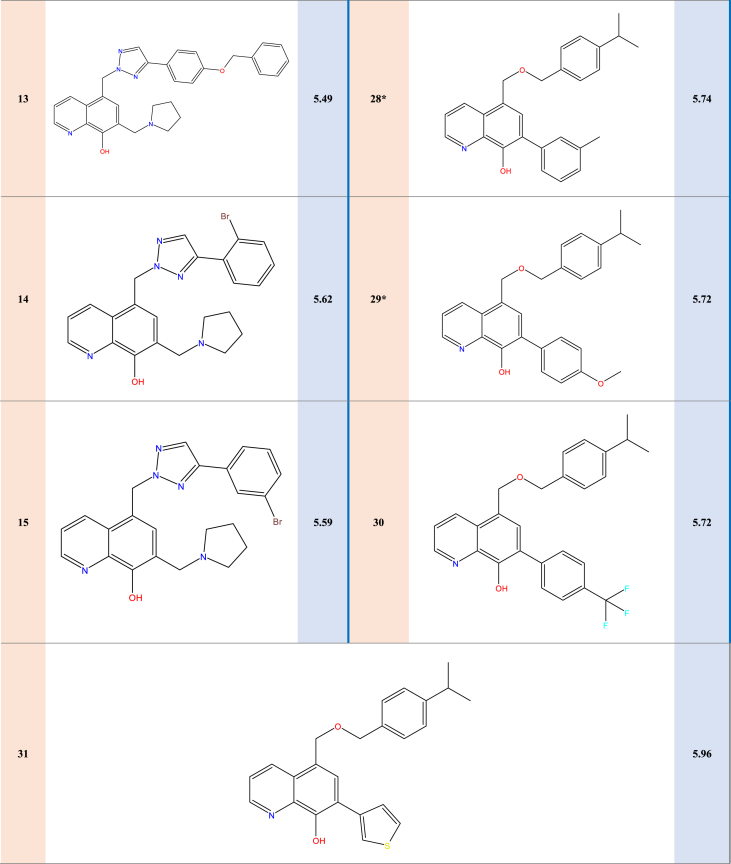
Structures and pIC50 values of novel selective survivin inhibitors derived from the hydroxyquinoline scaffold MX-106.

### 2.2 Examined compounds

Experimental data was used on survivin, a crucial member of the inhibitor of apoptosis (IAP) protein family, which we collected from a curated dataset that included 31 compounds that came from the previously synthesized MX-106 hydroxyquinoline scaffold to do molecular modeling [19]. To standardize and facilitate the analysis, we converted the observed activity values (IC_50_) into pIC_50_ values by using a logarithmic scale of log(IC_50_); these values are presented in Table 2.

**Table 2:** Descriptor results calculated for the 31 compounds with observed activities.

Comp	S	B	S-B	T	MW	NHBA	PSA	WI	MR	MV	IR	ST	D	E_HOMO_	E_LUMO_	ET	repul	pIC_50_
1	2.11	25.54	−0.13	−10.07	384.48	5.00	51.43	2312.00	115.28	304.40	1.68	52.70	1.26	−0.21	−0.07	−1223.54	2571.58	**5.05**
2∗	2.06	14.56	−0.05	−9.56	385.47	6.00	63.79	2312.00	113.72	293.00	1.70	55.90	1.31	−0.22	−0.06	−1239.56	2583.51	**5.30**
3	2.28	11.90	0.01	−4.80	374.48	4.00	45.06	2106.00	111.52	294.80	1.68	64.50	1.27	−0.21	−0.06	−1190.45	2439.83	**5.66**
4	2.48	7.66	0.18	−5.72	388.51	4.00	45.06	2339.00	116.15	310.90	1.67	62.90	1.24	−0.21	−0.06	−1229.78	2595.06	**5.98**
5	2.17	15.16	−0.01	−9.32	464.37	6.00	63.79	2573.00	121.28	305.50	1.72	58.50	1.51	−0.22	−0.07	−3813.10	3059.26	**5.51**
6	2.22	15.59	0.00	−5.45	453.47	9.00	63.79	3452.00	118.48	323.00	1.65	49.20	1.40	−0.22	−0.07	−1576.70	3137.93	**5.39**
7	2.44	15.65	0.03	−9.60	427.55	6.00	63.79	3157.00	127.18	339.40	1.67	49.90	1.25	−0.21	−0.06	−1357.54	2968.36	**5.34**
8	2.20	15.15	−0.03	−9.99	399.50	6.00	63.79	2573.00	118.15	308.20	1.69	53.20	1.29	−0.21	−0.06	−1278.89	2689.39	**5.70**
9	2.27	15.20	−0.02	−9.63	413.53	6.00	63.79	2864.00	122.76	324.20	1.68	52.10	1.27	−0.21	−0.06	−1318.21	2819.98	**5.49**
10	2.22	15.30	−0.03	−9.52	430.47	7.00	115.6	3157.00	119.38	298.30	1.73	65.00	1.44	−0.22	−0.10	−1444.12	2987.45	**5.39**
11	2.33	17.08	0.01	−9.54	415.50	7.00	73.02	2864.00	119.54	314.60	1.68	53.60	1.32	−0.21	−0.06	−1354.12	2863.61	**5.41**
12	2.06	14.52	−0.06	−9.62	410.48	7.00	87.58	2864.00	120.25	305.80	1.72	58.90	1.34	−0.22	−0.07	−1331.83	2735.52	**5.23**
13	2.71	17.28	0.02	−14.89	491.60	7.00	73.02	5102.00	144.83	383.20	1.68	53.40	1.28	−0.21	−0.06	−1585.22	3513.41	**5.49**
14	2.60	17.19	0.10	−8.38	464.37	6.00	63.79	2527.00	121.28	305.50	1.72	58.50	1.51	−0.21	−0.06	−3813.10	3275.71	**5.62**
15	2.18	15.02	0.00	−9.22	464.37	6.00	63.79	2550.00	121.28	305.50	1.72	58.50	1.51	−0.22	−0.07	−3813.10	3109.32	**5.59**
16	1.82	18.98	−0.16	1.09	391.49	6.00	63.79	2077.00	112.32	275.40	1.75	62.30	1.42	−0.21	−0.07	−1559.92	2586.39	**5.32**
17	2.13	12.75	0.19	−15.54	478.39	6.00	63.79	2827.00	125.89	321.60	1.71	56.90	1.48	−0.22	−0.06	−3852.30	3364.25	**5.66**
18	2.17	13.17	0.19	−11.65	467.50	9.00	63.79	3757.00	123.09	339.00	1.65	48.30	1.37	−0.22	−0.07	−1616.04	3314.93	**5.59**
19	2.47	15.73	0.40	−7.02	481.52	9.00	63.79	4065.00	127.69	355.10	1.64	47.60	1.35	−0.22	−0.07	−1655.35	3504.07	**5.51**
20	2.23	14.92	0.31	−12.24	455.49	9.00	63.79	3457.00	120.66	349.80	1.61	42.90	1.30	−0.21	−0.07	−1577.92	3192.63	**5.42**
21	2.21	14.43	0.33	−14.17	480.37	7.00	73.02	2827.00	122.67	312.00	1.72	58.80	1.53	−0.21	−0.07	−3888.33	3237.41	**5.62**
22∗	2.05	12.62	0.16	−18.70	479.38	7.00	75.82	2827.00	124.33	310.20	1.73	60.30	1.54	−0.22	−0.07	−3868.47	3240.09	**5.33**
23∗	1.77	3.58	0.17	−13.51	386.29	3.00	41.82	1509.00	101.72	281.30	1.64	51.60	1.37	−0.22	−0.07	−3553.77	2275.63	**5.68**
24	2.89	6.75	0.25	−18.82	383.49	3.00	41.82	2438.00	118.62	330.40	1.64	49.60	1.16	−0.21	−0.07	−1211.34	2466.55	**6.30**
25	2.95	6.14	0.21	−12.55	434.54	4.00	54.18	3352.00	134.56	357.70	1.68	55.10	1.21	−0.21	−0.07	−1381.05	3046.56	**6.22**
26	3.01	6.32	0.21	−10.36	434.54	4.00	54.18	3352.00	134.56	357.70	1.68	55.10	1.21	−0.22	−0.07	−1381.05	3050.10	**5.92**
27∗	2.91	7.00	0.26	−19.69	397.52	3.00	41.82	2684.00	123.45	346.70	1.63	48.40	1.15	−0.20	−0.06	−1250.66	2607.52	**5.74**
28∗	2.96	6.89	0.25	−19.44	397.52	3.00	41.82	2661.00	123.45	346.70	1.63	48.40	1.15	−0.20	−0.06	−1250.66	2617.33	**5.74**
29∗	3.09	9.34	0.32	−19.22	413.52	4.00	51.05	2960.00	125.30	354.40	1.62	48.10	1.17	−0.20	−0.06	−1325.89	2760.51	**5.72**
30	2.91	7.39	0.28	−15.14	451.49	6.00	41.82	3518.00	123.60	363.90	1.59	43.40	1.24	−0.22	−0.07	−1548.48	3095.35	**5.72**
31	2.42	11.39	0.19	−13.48	389.51	3.00	41.82	2218.00	117.01	319.90	1.65	52.20	1.22	−0.20	−0.07	−1532.10	2489.62	**5.96**

**S**: Stretch energy (kcal/mol), **B**: Bend energy (kcal/mol), **S-B**: Stretch-Bend energy (kcal/mol), **T**: Torsion energy (kcal/mol), **NHBA**: number of hydrogen bond acceptors, **Mw**: Molecular weight (g/mol), **PSA**: Polar Surface Area, **WI**: Wiener Index, **MR:** Molar Refractivity index, **MV**: Molar Volume (cm^3^)**, IR**: Index of Refraction, **ST**: Surface Tension (dyne/cm), **E**_**T**_: total energy (Hartree), **D:** Density (g/cm^3^), **repul**: repulsion energy (Hartree), **E**_**HOMO**_**:** Energy of the Highest occupied molecular orbital (Hartree)**, E**_**LUMO**_**:** Energy of the lowest unoccupied molecular orbital (Hartree).

### 2.3 Descriptor calculation for studied compounds

To construct a dependable QSAR model, a comprehensive set of 30 molecular descriptors that encompassed factors that related to lipophilicity, geometric properties, physicochemical attributes, and structural effects was calculated as presented in Fig. S1. These molecular properties were determined using a molecular mechanics method (MM2) that is implemented in software packages such as ACD/ChemSketch [20] and ChemBioOffice [21]. Molecular geometry was optimized with the assistance of density functional theory (DFT) calculations with the B_3_LYP functional and 6-311G(d,p) basis set [22,23]. Electronic properties were subsequently computed utilizing the Gaussian 09 software [24]. To reduce the size of calculated descriptors, the principal component analysis (PCA) method was applied based on Pearson correlation matrix, as resulted in Fig. S2, in which the molecular descriptors strongly correlated having Pearson correlation coefficients superior than 0.9 were removed and 17 uncorrelated descriptors were retained. The results of poorly correlated descriptors are shown in Table 2.

### 2.4 Quantitative structure-activity relationship modeling

We selected 31 compounds from previous studies that exhibited selective survivin inhibitory activity and were derived from the hydroxyquinoline scaffold MX-106. The data set was randomly split into a training set of 25 molecules for model development and a test set of six molecules to evaluate their robustness. Multiple linear regression (MLR) and artificial neural networks (ANNs) were employed to construct the QSAR model [25].

### 2.5 Multiple linear regression

MLR is commonly used in many QSAR studies due to its simplicity and reliability in selecting molecular descriptors [26]. Additionally, MLR is combined with ANN and multiple nonlinear regression (MNLR) techniques to identify suitable descriptors that can be employed as input factors for constructing QSAR models. MLR model is based on a linear relationship between the dependent variable and specific independent descriptors:

Eq. 1 $$Y=a_{0}+\sum_{i=1}^{n}a_{i}X_{i}$$

Where *n* represents the number of molecular descriptors, *Y* denotes the dependent variable, and *X*_*i*_ signifies the independent variable; ***a***_***0***_ represents the constant component of Equation (1), and ***a***_***i***_ represents the molecular descriptor coefficients.

### 2.6 MLR-QSAR modeling efficiency

#### 2.6.1 Multiple nonlinear regression

choosing the predicted model that captures the nonlinear relationship between the biological activity (*Y*) with respect to the calculated descriptors (*Xi*) can be achieved through various nonlinear regression techniques [27]. For this study, we employed a second-order polynomial model to build a QSAR model while using a nonlinear regression approach. The following equation describes the nonlinear variation that links the studied activity and computed descriptors:

Eq. 2 $$Y=a_{0}+\sum_{i=1}^{n}a_{i}{\times X}_{i}+b_{i}\times X_{i}^{2}$$

The equation may be recast as follows in relation to the MLR approach for QSAR studies:

The dependent variable, which is denoted by *Y* in Equation (2), represents the biological activity that has to be anticipated. The molecular descriptors are included in the independent variables, *X*_*i*_, and their number is denoted with *n*. The established equation also contains a constant value, denoted as ***a***_***0***_, as well as the factors ***a***_***i***_ and ***b***_***i***_, which represent the factors associated to each descriptor at the equation.

#### 2.6.2 Artificial neural networks

**Fig. 1:**
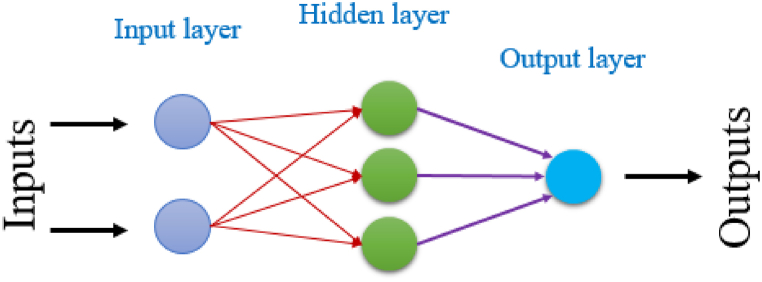
ANN model architecture.

We utilized ANNs to enhance compound characterization and establish a predictive model that links the calculated descriptors by the previous MLR model with observed activity values. This ANN-based QSAR model validated the significance of the selected molecular descriptors and accurately predicted activity for each molecule [28]. The ANN model that was developed for this study follows a feed-forward approach by employing sigmoid and linear transfer functions in the hidden and output layers, respectively. The ANN architecture deployed in this study is make up of three layers of neurons, referred to as the input, hidden and output layers, as shown in Fig. 1.

To certify the statistical validity of the ANN model and the accuracy of predictions, the number of neurons in the input layer must not exceed the number of molecular descriptors used in the MLR model. The output layer provides the predicted activity values. To determine the optimal number of hidden neurons, we calculated the ρ parameter, that is defined as the ratio of the number of weights to the number of connections. The parameter ρ should ideally fall between 1 and 3 to ensure the statistical acceptability of the ANN model and accurate predictions while considering the contribution of all elements within the database [29].

#### 2.6.3 Leave-one-out cross-validation

To test the precision of the QSAR models developed using MLR, MNLR and ANN techniques, we applied a no-intervention cross-validation (LOO-CV) test [30]. In LOO-CV, one molecule is sequentially removed from the dataset, and the model is retrained on the remaining molecules to predict the removed molecule's activity [26]. This process is repeated for all molecules, and the coefficient of performance $R_{cv}^{2}$ is calculated as described in Equation (3). This approach helps evaluate the model's predictive power and generalizability [30]. A resilient model for internal predictions typically exhibits an $R_{cv}^{2}$ value that is greater than 0.5.

Eq. 3 $$R_{\text{cv}}^{2}=1-\frac{\sum \left(Y_{\text{ob}}\left(trai\right)-Y_{\text{ca}}\left(trai\right)\right)²}{\sum \left(Y_{\text{Ob}}\left(trai\right)-\bar{Y_{trai}}\left(trai\right)\right)²}$$

$Y_{ob}\left(trai\right)$ is a reference to the training set's actual observed response value. $Y_{ca}\left(trai\right)$ indicates the response value that the training set's LOO-CV method predicted, and $\bar{Y_{trai}}\left(trai\right)$ represents the value of observed and predicted responses averaged over the training set.

#### 2.6.4 *Y*-randomization test

*Y*-randomization test is used to avoid unintended correlations between the selected descriptors and their activities in the model initially obtained using the MLR technique. Any chance relationship between molecular descriptors (*X* values) and inhibitory activity (*Y* values) could compromise the effectiveness and validity of not only the MLR model but also the MNLR and ANN models. During the *Y*-randomization test, experimental properties/activity values were randomly reassigned to the descriptors of the original model, which resulted in the creation of new models [31]. The QSAR model's validity was confirmed by Y-randomization testing. The average random correlation coefficient $\left(R_{r}^{2}\right)$ of the randomly generated models was significantly lower than the correlation coefficient $\left(R_{r}^{2}\right)$ of the original, non-random model, indicating that the model's performance was not due to chance [32].

### 2.7 Applicability domain

The applicability domain process was used to assess the predictive capability of the developed QSAR model and identify outliers within the training and test sets. Its use involved calculating the diagonal elements (leverage values) of the hat matrix, which is also known as the projection matrix [33,34]. The hat matrix (7373) was calculated by projecting the observations (*Y*) on predicted values $\left(\hat{Y}\right)$ using the following equation:

Eq. 4 $$h_{i}=x_{i}{\left(X^{T}X\right)}^{-1}x_{i}^{T}\;i=1,2,\ldots ‥,n$$

The hat matrix is a mathematical tool that is used to assess the influence of each compound on a model's predictions. It considers a compound's list of molecular properties (descriptors) relative to all other compounds in the training set [35]. The diagonal elements of this matrix, which are called leverage values, indicate how much a specific compound's properties affect the predicted values of others. These leverage values were used to define the applicability domain of the model and identify any unusual compounds in the dataset. Consequently, the residuals (the differences between predicted and observed values) from both the training and test sets were analyzed in relation to the leverage values that were extracted from the corresponding hat matrix.

### 2.8 Analysis of the molecular electrostatic potential

Molecular electrostatic potential (MEP) is a method that utilizes DFT calculations based on the B3LYP-6-311G(d,p) basis set to visually represent the obtained results [[36], [37], [38]]. The MEP method's ability to visualize the chemical reactivity of atoms and active centers makes it highly significant. The MEP analysis provides insights into hydrogen bonding and the biological reactivity of chemical compounds' electrophilic and nucleophilic sites. By employing the colors red, orange, yellow, green, and blue, the MEP diagram illustrates the variation in electrostatic potentials in an ascending order of electrostatic potential (red < orange < yellow < green < blue). Furthermore, the chemical potential's value increases in this direction. Blue indicates nucleophilicity, while red and orange indicate the potential for electrophilicity [39,40].

### 2.9 Molecular docking simulations

Molecular docking technology has taken on considerable importance in the field of drug discovery [41,42]; we have worked in this part to explore the mechanism of the inhibition of the molecules under investigation toward the protein target [43] in line with the standard protocol for preparing ligands and proteins, as declared in the literature [[44], [45], [46]], by taking several steps into account. First, three-dimensional structures of targeted proteins were extracted from the RCSB protein data bank (PDB) (https://www.rcsb.org/). Second, the proteins in PDB formats were prepared utilizing Discovery Studio (2021) software [47]; this was accomplished by deleting all water molecules, and suspended ligands and adding the Gasteiger charges [48,49]. Third, the studied ligands and the prepared receptors were changed from PDB to PDBQT formats utilizing the Autodock software [50]. Finally, grid boxes encompassing the entire targeted protein were generated using a spacing of 0.375 Å along each axis (X, Y, and Z). The resulting protein-ligand complexes were then visualized in Discovery Studio 2021 software [51,52].

### 2.10 Molecular dynamics simulation

Hydroxyquinoline derivatives 24, 25, pred2 and pred6 compounds have been chosen to build the MD simulations based on their docking scores against human survivin. MD simulation was executed for each of the macromolecular complexes of human survivin with hydroxyquinoline derivatives for a period of 100 ns by using the Desmond module of Schrodinger's Maestro software [[53], [54], [55], [56], [57], [58]]. Explicit solvent molecules were added, which was followed by their neutralization through the addition of the oppositively charged ions. The macromolecular system was relaxed by applying steepest-descent algorithm for energy minimization to avoid the possibilities of stearic clashes as well as poor intramolecular contacts. The macromolecular system was equilibrated by considering the low temperature with constant pressure (NPT) for carrying out the simulations by applying positional constraints for gradual rise in the temperature for making the system balanced and stable for carrying out the simulation [[59], [60], [61]]. Position of each of the atoms, their RMSD values and the system's energy was considered while performing the 100 ns simulation with appropriate results concluding the dynamic behavior of the macromolecular system with long-term insights into their functional as well as structural stability [[62], [63], [64], [65], [66], [67], [68], [69]].

### 2.11 In silico pharmacokinetic-Pharmacodynamic modeling (ADMET)

Advancements in computational techniques have stimulated the discovery of novel drug candidates, minimizing the need for time-consuming experiments and increasing success rates. This has led to the prioritization of the early identification of ADMET properties and drug similarities in the initial stages of drug discovery. A prominent method for assessing these key ADMET properties, is utilized through in silico research. This computational approach offers advantages such as reduced costs and faster development times, which make it a valuable tool in modern drug discovery [70]. Drug development involves several processes: absorption (the substances uptake in the human intestine), distribution and chemical transformation of substances throughout the body, and excretion (the removal of substances). Evaluating a substance's toxicity is also crucial.

These standards were created by Ghose, Veber, Egan, Lipinski, and Muegge to estimate how drugs interact with other molecules. Evaluating the ADME properties of pharmaceuticals is crucial for human use, and the guidelines of Lipinski, Veber, and Egan are especially useful. Based on the oral bioavailability of small compounds and their 2D structures, these guidelines assist in identifying potential drug candidates [71]. Compounds that violate two or more Lipinski, Veber, and Egan (LVE) rules can exhibit significantly altered pharmacokinetic ADMET properties. Despite this, nearly 10 % of drugs that progress to clinical trials violate LVE rules. To address this, we also took the topological polar surface area (TPSA) and the number of rotational bonds (n-ROTB) into account [72]. These parameters helped us predict whether the compound interacts with receptors flexibly or rigidly.

## 3 Results and discussion

### 3.1 Multiple linear regressions

After obtaining information on the chemical descriptors of 31 compounds, we undertook many efforts to construct a trustworthy model (see Table 2). Only two stretch descriptors, **Bend** and **E**_**HOMO**_, were used to build the best QSAR model. The following molecules (2, 22, 23, 27, 28, and 29) were chosen for the whole test on the basis of the results. The following molecules (1, 3, 4, 5, 6, 7, 8, 9, 10, 11, 12, 13, 14, 15, 16, 17, 18, 19, 20, 21, 23, 24, 25, 26, 30, and 31) were chosen for the entire training. below depicts the QSAR model that we created using the MLR approach:

Eq. 4 $$\mathrm{pI}C_{50}==10.65279-0.05759\times B+19.87623\times E_{\mathrm{HOMO}}$$

$$N=26\;;\;R=0.925;\;R^{2}=0.855;\;R_{Ajus}^{2}=0.842;\;MSE=0.014;\;F=64.896;\mathrm{Pr}<\;0.\;0001;\;R_{cv}^{2}=0.\;82$$

*N* denotes the compound number in the training set, and *MSE* denotes the root mean square error.

Survivin-selective biological inhibitory activity (pIC_50_) values were linearly associated with the two chosen descriptors, as depicted in. The following parameters were used to evaluate a QSAR model that was created using the MLR technique: *R*^*2*^, *F*, *MSE*, the *P*-value, and ${\;R}_{cv}^{2}$.

**Fig. 2:**
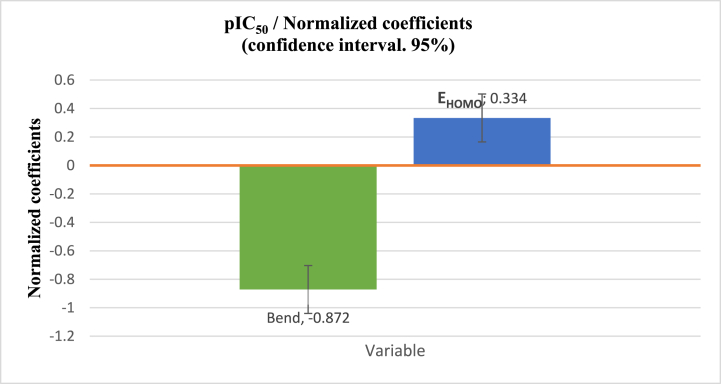
Contribution coefficients of two calculated descriptors involved in the MLR equation.

**Fig. 3:**
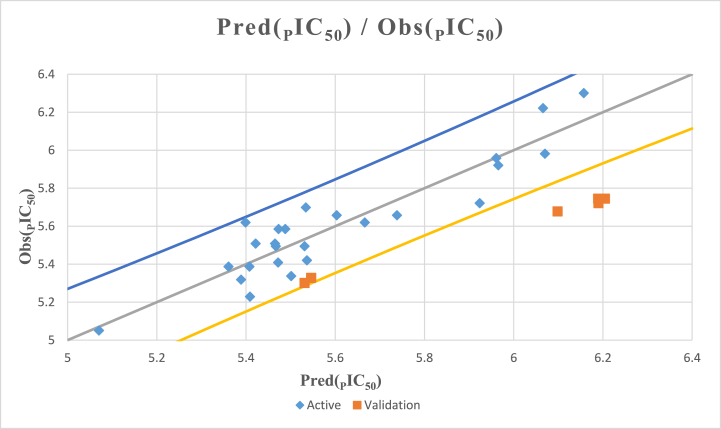
MLR correlation between predicted and observed activity values.

The statistical measures, including a high coefficient of determination (*R*^*2*^ = 0.855), a low mean squared error (*MSE* = 0.014), and a high *F*-statistic (*F* = 64.896), indicate that the QSAR model presented in Equation (5) performs well statistically. These results suggest that the model can accurately capture the relationship between molecular properties and biological activity. Moreover, the calculated *P*-value, which was inferior to 0.05 (*Pr* < 0.0001), signifies that the QSAR model exhibits statistical significance at a confidence interval exceeding 95 %. The energy of the highest occupied molecular orbital (E_HOMO_) had a positive impact on biological activity, while bend exerted a negative influence, as illustrated in Fig. 2. Furthermore, the cross-validation correlation coefficient ($R_{cv}^{2}$ = 0.82), which significantly exceeded the threshold of 0.5, attests to the robustness of the QSAR model that was established via the MLR technique. The value of $R_{cv}^{2}$, which was lower than *R*^*2*^, indicates the model's sensitivity when an element is omitted from the training set. The link between the observed and expected activity levels is depicted in Fig. 3. Both training and test set compounds were successfully captured in the MLR model. Fig. 3 visually demonstrates the strong correlation between the observed and predicted pIC_50_ values, which were substantiated using the low MSE value. Equation (5) includes the two descriptors that exhibit a pronounced linear correlation with biological activity, namely pIC_50_. To improve the alignment between the activities that were projected by the MLR model and the two calculated descriptors (*B* and E_HOMO_), a new predicted model was formulated based on two nonlinear methodologies: nonlinear multiple regression (NLMR) and ANN techniques.

### 3.2 Multiple nonlinear regression

Nonlinear QSAR model generated with the MNLR technique is presented in:

Eq. 5 $$\mathrm{pI}C_{50}=7.73241\;–\;0.05702\times B\;–\;7.53421\times E_{\text{HOMO}}\;–\;0.00002\times B^{2}\;–\;64.36113\times E_{\text{HOMO}}^{2}$$

$$N=25;\;R=0.92;\;R^{2}=0.855;\;MSE=0.015$$

The non-linear QSAR model performance indicators obtained, namely *R* = 0.92, *R*^*2*^ = 0.855, and *MSE* = 0.015, provide clear evidence that this model is statistically valid. The following tables illustrate the inhibitory activities that were estimated and observed using the generated QSAR model that was created using the test and training sets for the two nonlinear and linear models (Table 3).

**Table 3:** Observed and predicted activities by the QSAR model developed based on the test and training sets.

Comp	B	E_HOMO_	pIC_50_Obs	MLR		MNLR	
				pIC_50_Pred	Resid	pIC_50_Pred	Resid
1	25.538	−0.20686	5.05	5.070	−0.020	5.067	−0.016
2∗	14.5555	−0.2155	5.30	5.531	−0.230	5.533	−0.232
3	11.8958	−0.21278	5.66	5.738	−0.081	5.740	−0.083
4	7.6584	−0.20837	5.98	6.070	−0.089	6.070	−0.088
5	15.1575	−0.21709	5.51	5.465	0.044	5.466	0.043
6	15.5949	−0.2187	5.39	5.408	−0.020	5.407	−0.020
7	15.6517	−0.21383	5.34	5.501	−0.164	5.503	−0.166
8	15.1486	−0.21364	5.70	5.534	0.165	5.536	0.163
9	15.2017	−0.21363	5.49	5.531	−0.036	5.533	−0.038
10	15.2955	−0.22193	5.39	5.361	0.026	5.357	0.030
11	17.0817	−0.21116	5.41	5.472	−0.063	5.473	−0.064
12	14.5183	−0.22176	5.23	5.409	−0.180	5.406	−0.177
13	17.2789	−0.21085	5.49	5.467	0.028	5.468	0.027
14	17.1924	−0.21452	5.62	5.399	0.221	5.400	0.219
15	15.0218	−0.21708	5.59	5.473	0.112	5.474	0.111
16	18.9751	−0.20985	5.32	5.389	−0.070	5.390	−0.071
17	12.751	−0.2171	5.66	5.603	0.054	5.604	0.053
18	13.1711	−0.22169	5.59	5.488	0.097	5.485	0.100
19	15.7338	−0.2176	5.51	5.422	0.087	5.422	0.087
20	14.9207	−0.21417	5.42	5.537	−0.116	5.538	−0.118
21	14.4285	−0.20907	5.62	5.666	−0.046	5.667	−0.047
22∗	12.6154	−0.22037	5.33	5.546	−0.218	5.544	−0.217
23∗	3.58	−0.21877	5.68	6.098	−0.420	6.096	−0.418
24	6.75	−0.20662	6.30	6.157	0.144	6.156	0.145
25	6.1379	−0.213	6.22	6.066	0.156	6.066	0.155
26	6.3217	−0.21751	5.92	5.965	−0.045	5.965	−0.044
27∗	7.0005	−0.20351	5.74	6.205	−0.460	6.200	−0.455
28∗	6.8851	−0.20461	5.74	6.189	−0.445	6.186	−0.441
29∗	9.3373	−0.19747	5.72	6.190	−0.469	6.176	−0.455
30	7.3917	−0.2165	5.72	5.924	−0.203	5.924	−0.203
31	11.386	−0.20306	5.96	5.961	−0.002	5.957	0.002

To further assess the model's reliability, we utilized a LOO-CV technique. When a LOO-CV is performed, each data point is excluded one at a time, and the model is rebuilt to predict the excluded point's activity. The high coefficient of determination ($R_{cv}^{2}$) that is obtained using the LOO-CV (0.82) indicates the model's robustness. Our result suggests that all 25 training set compounds played a role in model development by contributing to its effectiveness and reliability.

**Fig. 4:**
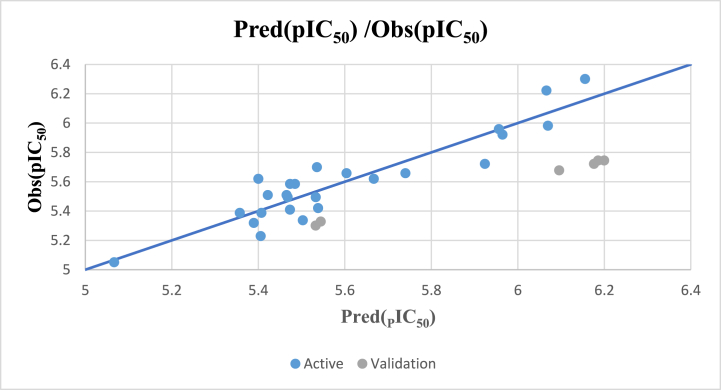
Correlations between the observed and predicted activity using the MNLR model.

Fig. 4 illustrates the good correlation between the experimental pIC_50_ values and the values that were predicted using the MNLR technique. This uniform distribution of the residuals confirms the absence of systematic bias in the developed QSAR model.

### 3.3 Artificial neural networks

When creating a QSAR model with the ANN approach, we employed a 2-3-1 architecture with a 1.92 ρ parameter. By using a ρ value that ranged from 1 to 3, we observed that the hidden layer's 3 was directly proportional to the input layer's 2 descriptors, which enabled the output layer's 1 to anticipate the pIC_50_ values. By using a very high coefficient of determination (*R*^*2*^) of 0.92, the QSAR model that was built using the ANN approach demonstrates a very strong match to the data. Furthermore, the MSE was low it was only 0.15 which indicates that the model's predictions are highly accurate.

Our findings indicate that the QSAR model is statistically significant for predicting the activity of selective survivin inhibitors derived from the MX-106 hydroxyquinoline scaffold. Notably, the model utilizes descriptors (**B** and **E**_**HOMO**_) that were chosen for their suitability in this context. This model allowed us to estimate pIC_50_ values using these relevant descriptors.

**Fig. 5:**
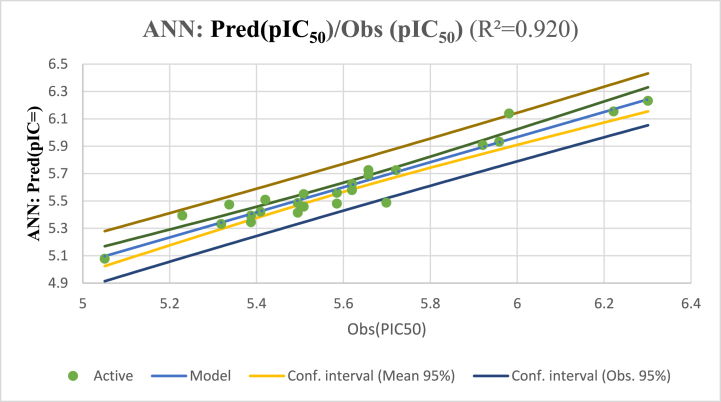
The correlation between the observed and the predicted activities calculated using the ANN model.

The training set's candidate pIC_50_ values were uniformly distributed, as depicted in Fig. 5, which guaranteed that the ANN model's predictions would closely resemble the pIC_50_ values that have been found in experiments.

### 3.4 Leave-one-out cross-validation

**Fig. 6:**
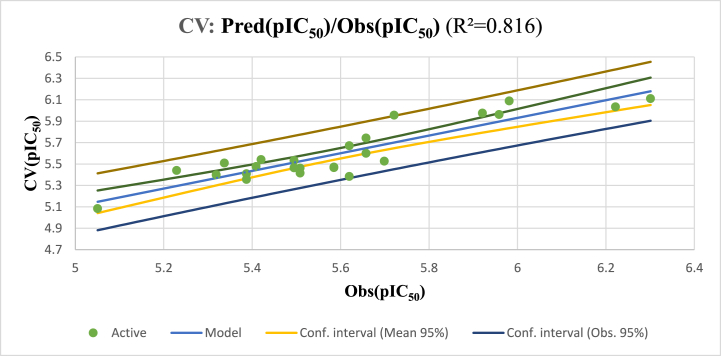
Correlation between observed and predicted activities calculated using LOO-CV.

These results demonstrate the stability and robustness of the proposed QSAR model, as evidenced by the high *R*^*2*^ and lowest *RMSE* that were obtained during a LOO-CV (Fig. 6). The model's performance remained consistent even when individual data points were left out during the validation process. It is important to acknowledge that while cross-validation is a valuable tool for assessing model performance, it may not fully capture a model's generalizability for entirely new datasets.

### 3.5 *Y*-randomization test

In QSAR investigations, randomization is frequently used to guarantee the resilience and dependability of the resulting models. Randomization is used to evaluate possible correlations and validate the regression model that has been chosen. In this instance, *Y*-randomization was applied to evaluate the model's efficacy. The approach involved randomly redistributing and jumbling the inhibitory activity values of the studied compounds before repeatedly recreating the model using the original descriptors. This procedure was carried out 100 times to gain a thorough assessment (see Table 4).

**Table 4:** Y-Randomization test results.

*Model*	*R*_*r*_	$R_{r}^{2}$	$R_{r.cv}^{2}$	*Model*	*R*_*r*_	$R_{r}^{2}$	$R_{r.cv}^{2}$
*Original*	***0.924698***	***0.855066***	***0.815471***	**Rnd51**	**0.32602**	**0.106289**	**−0.1616**
Rnd1	**0.308317**	**0.095059**	**−0.37395**	**Rnd52**	**0.10845**	**0.011761**	**−0.23477**
Rnd2	**0.216723**	**0.046969**	**−0.19821**	**Rnd53**	**0.230563**	**0.053159**	**−0.23854**
Rnd3	**0.101777**	**0.010359**	**−0.39777**	**Rnd54**	**0.428943**	**0.183992**	**−0.14408**
Rnd4	**0.315666**	**0.099645**	**−0.2361**	**Rnd55**	**0.276117**	**0.076241**	**−0.15082**
Rnd5	**0.131026**	**0.017168**	**−0.29318**	**Rnd56**	**0.466581**	**0.217697**	**0.093853**
Rnd6	**0.035305**	**0.001246**	**−0.3089**	**Rnd57**	**0.527833**	**0.278607**	**0.094106**
Rnd7	**0.488318**	**0.238455**	**−0.04462**	**Rnd58**	**0.090367**	**0.008166**	**−0.26141**
Rnd8	**0.135049**	**0.018238**	**−0.22969**	**Rnd59**	**0.208787**	**0.043592**	**−0.2011**
Rnd9	**0.031932**	**0.00102**	**−0.39183**	**Rnd60**	**0.382748**	**0.146496**	**−0.16994**
Rnd10	**0.588213**	**0.345994**	**0.198903**	**Rnd61**	**0.279599**	**0.078176**	**−0.1521**
Rnd11	**0.210656**	**0.044376**	**−0.17538**	**Rnd62**	**0.17246**	**0.029742**	**−0.32009**
Rnd12	**0.310209**	**0.096229**	**−0.16853**	**Rnd63**	**0.258446**	**0.066794**	**−0.21925**
Rnd13	**0.487868**	**0.238015**	**−0.0123**	**Rnd64**	**0.342039**	**0.116991**	**−0.27141**
Rnd14	**0.636377**	**0.404975**	**0.19603**	**Rnd65**	**0.26465**	**0.070039**	**−0.19548**
Rnd15	**0.367367**	**0.134959**	**−0.05875**	**Rnd66**	**0.130933**	**0.017143**	**−0.28405**
Rnd16	**0.229082**	**0.052479**	**−0.17554**	**Rnd67**	**0.15205**	**0.023119**	**−0.28467**
Rnd17	**0.238679**	**0.056968**	**−0.23221**	**Rnd68**	**0.189306**	**0.035837**	**−0.27446**
Rnd18	**0.310453**	**0.096381**	**−0.15507**	**Rnd69**	**0.237885**	**0.056589**	**−0.19936**
Rnd19	**0.159098**	**0.025312**	**−0.2179**	**Rnd70**	**0.516103**	**0.266362**	**0.060648**
Rnd20	**0.339056**	**0.114959**	**−0.1249**	**Rnd71**	**0.176707**	**0.031225**	**−0.36705**
Rnd21	**0.309638**	**0.095875**	**−0.17452**	**Rnd72**	**0.059808**	**0.003577**	**−0.33145**
Rnd22	**0.286855**	**0.082286**	**−0.19467**	**Rnd73**	**0.183352**	**0.033618**	**−0.30988**
Rnd23	**0.508294**	**0.258362**	**0.050619**	**Rnd74**	**0.053201**	**0.00283**	**−0.15129**
Rnd24	**0.140953**	**0.019868**	**−0.34271**	**Rnd75**	**0.206752**	**0.042746**	**−0.3253**
Rnd25	**0.055972**	**0.003133**	**−0.32234**	**Rnd76**	**0.201262**	**0.040507**	**−0.28828**
Rnd26	**0.083036**	**0.006895**	**−0.39099**	**Rnd77**	**0.210404**	**0.04427**	**−0.36889**
Rnd27	**0.336932**	**0.113523**	**−0.13347**	**Rnd78**	**0.26923**	**0.072485**	**−0.13237**
Rnd28	**0.293099**	**0.085907**	**−0.20692**	**Rnd79**	**0.225124**	**0.050681**	**−0.19184**
Rnd29	**0.409262**	**0.167495**	**−0.1181**	**Rnd80**	**0.303961**	**0.092392**	**−0.12299**
Rnd30	**0.129531**	**0.016778**	**−0.35015**	**Rnd81**	**0.138099**	**0.019071**	**−0.22659**
Rnd31	**0.094583**	**0.008946**	**−0.20384**	**Rnd82**	**0.208181**	**0.04334**	**−0.2566**
Rnd32	**0.402449**	**0.161965**	**−0.06169**	**Rnd83**	**0.278025**	**0.077298**	**−0.12898**
Rnd33	**0.309888**	**0.096031**	**−0.07975**	**Rnd84**	**0.242069**	**0.058597**	**−0.41427**
Rnd34	**0.473259**	**0.223974**	**−0.05777**	**Rnd85**	**0.29404**	**0.08646**	**−0.1138**
Rnd35	**0.275741**	**0.076033**	**−0.12479**	**Rnd86**	**0.302074**	**0.091249**	**−0.15787**
Rnd36	**0.385348**	**0.148493**	**−0.10587**	**Rnd87**	**0.391913**	**0.153596**	**−0.0451**
Rnd37	**0.081786**	**0.006689**	**−0.2925**	**Rnd88**	**0.273474**	**0.074788**	**−0.31258**
Rnd38	**0.387769**	**0.150365**	**−0.14343**	**Rnd89**	**0.343768**	**0.118176**	**−0.0986**
Rnd39	**0.043873**	**0.001925**	**−0.28504**	**Rnd90**	**0.106722**	**0.01139**	**−0.27818**
Rnd40	**0.280096**	**0.078454**	**−0.4029**	**Rnd91**	**0.251151**	**0.063077**	**−0.13837**
Rnd41	**0.258212**	**0.066674**	**−0.20881**	**Rnd92**	**0.300393**	**0.090236**	**−0.13606**
Rnd42	**0.146773**	**0.021542**	**−0.19367**	**Rnd93**	**0.05793**	**0.003356**	**−0.24703**
Rnd43	**0.279027**	**0.077856**	**−0.19061**	**Rnd94**	**0.128125**	**0.016416**	**−0.20908**
Rnd44	**0.344799**	**0.118886**	**−0.11534**	**Rnd95**	**0.245909**	**0.060471**	**−0.20736**
Rnd45	**0.301548**	**0.090931**	**−0.10938**	**Rnd96**	**0.097873**	**0.009579**	**−0.30084**
Rnd46	**0.208342**	**0.043407**	**−0.25046**	**Rnd97**	**0.337511**	**0.113913**	**−0.11193**
Rnd47	**0.249807**	**0.062404**	**−0.18148**	**Rnd98**	**0.30241**	**0.091452**	**−0.216**
Rnd48	**0.476843**	**0.227379**	**−0.06969**	**Rnd99**	**0.420043**	**0.176436**	**−0.02849**
Rnd49	**0.435992**	**0.190089**	**−0.10974**	**Rnd100**	**0.189894**	**0.03606**	**−0.25539**
Rnd50	**0.148301**	**0.021993**	**−0.30831**				
**Rnd Modes Parameters**							
Average *R*_*r*_:	**0.261685**						
Average $R_{r}^{2}$	**0.085591**						
Average $R_{r.cv}^{2}$:	**−0.19065**						
_c_$R_{p}^{2}$ :	**0.820112**						

For these randomized models, the average correlation coefficients were *R* = 0.26, $R^{2}$ = 0.085, and ${Q_{cv}}^{2}$ = 0.82. It is evident from comparing these averages to the model's results that the random values' aims resulted in noticeably lower $R^{2}$ and ${Q_{cv}}^{2}$ averages. These findings demonstrate that the model's observed correlations between the activities and descriptors were not supported, which confirms the model's robustness. As a result, this randomization test validates the model's predictive power and reliability.

### 3.6 Applicability domain

**Fig. 7:**
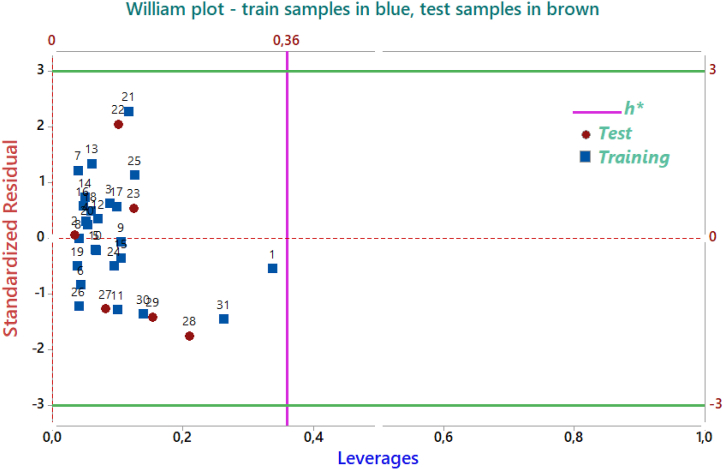
The Williams graph of the model presented in Equation 5.

To examine the AD of the MLR-QSAR model, a Williams plot was created, as illustrated in Fig. 7.

The Williams plot (Fig. 7) illustrates how a leverage analysis was used to determine the AD of the QSAR model. The findings of the William plots demonstrate that, for every compound in the training and test sets, the leverage values were less than the warning leverage, which is represented by the formula *h*∗ = 0.36 as (*h*∗ = 3 × (*K* + 1)/*n*, where *K* is the number of model parameters and *n* is the number of compounds.

The absence of outliers in the test sets allowed the QSAR model to make an accurate forecast. As a result, every tested chemical fell inside the AD, which demonstrates the validity of the activity levels that were anticipated.

### 3.7 External validation

Internal and external validations were employed to evaluate the models' significance and exact predictive power [73].

**Fig. 8:**
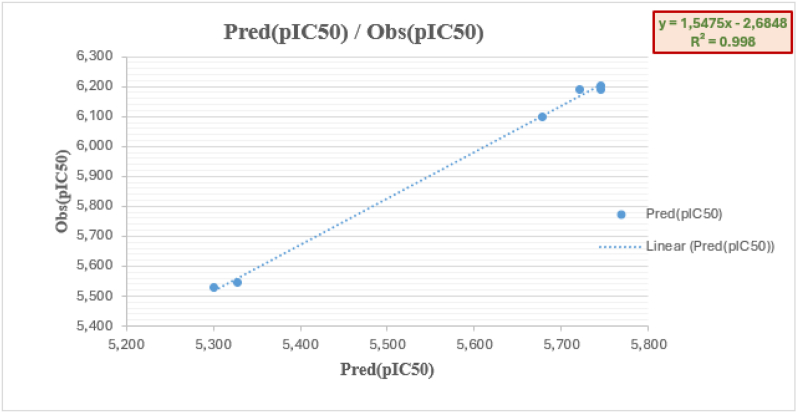
Observed correlations between the predicted and observed activities using the MLR model for the test set.

An $R_{cv}^{2}$ of 0.81 was found for LOO-CV coefficients in the best model. A test set of six substances, which produced a correlation coefficient of determination $R_{test}^{2}$ of 0.998 (Fig. 8), confirmed the prediction of the model. Table 3 displays the expected molecular activity levels for the training and model validation sets.

### 3.8 Molecular docking analysis

**Fig. 9:**
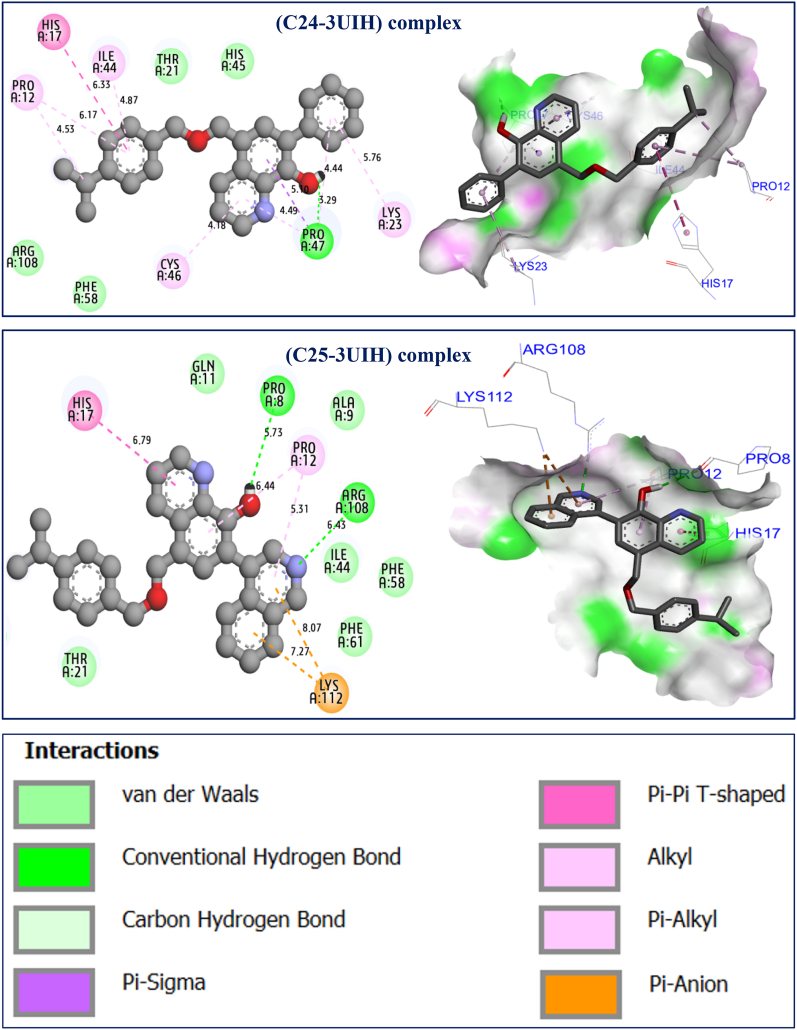
2D and 3D visualizations of intermolecular interactions between the human surviving protein (3UIH.pdb) and the most active ligands (C24 and C25), respectively.

**Fig. 10:**
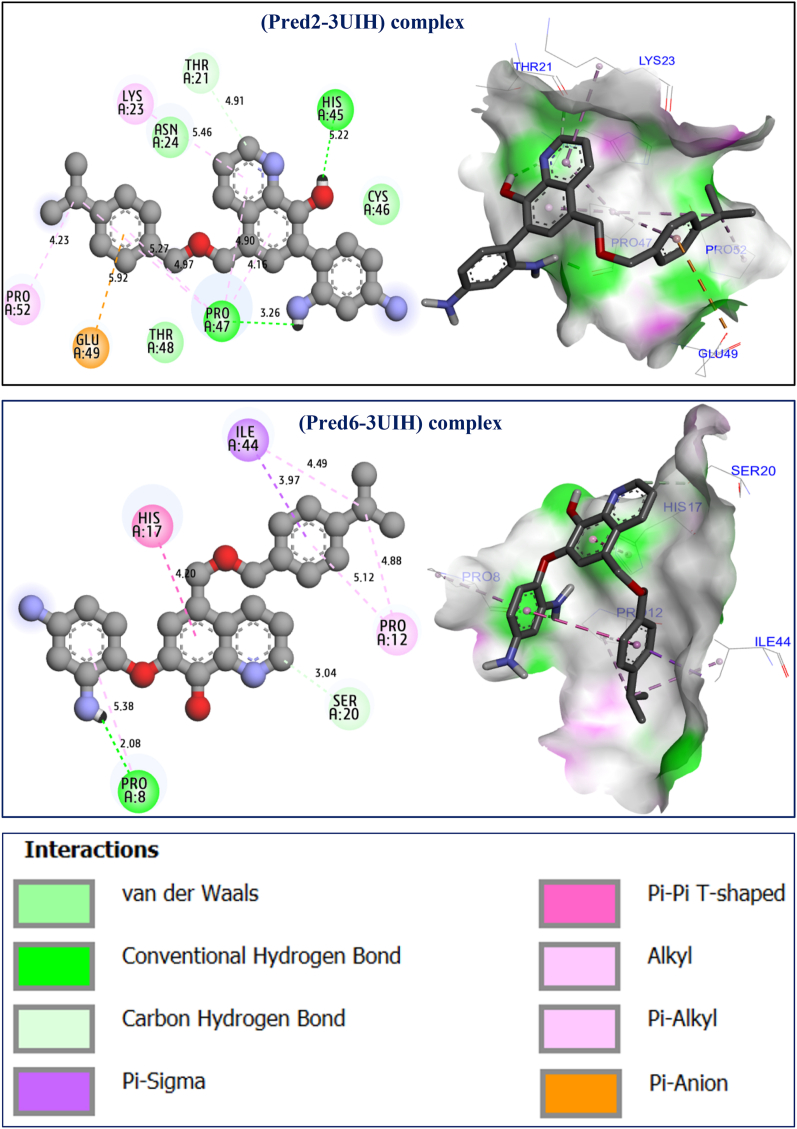
2D and 3D visualizations of intermolecular interactions between the human surviving protein (3UIH.pdb) and the predicted ligands labeled Pred2 and Pred6, respectively.

**Fig. 11:**
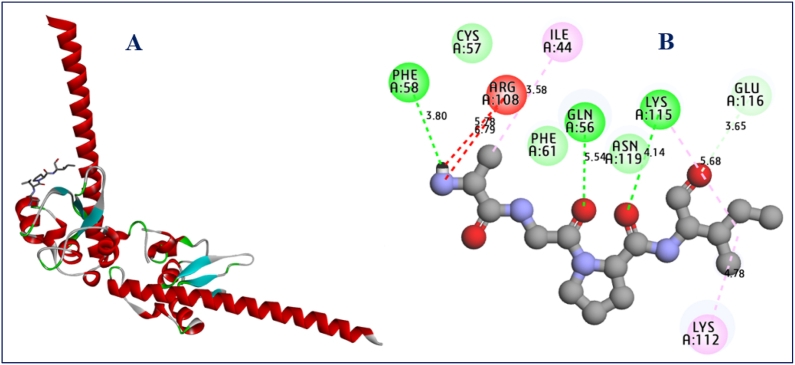
Crystal structure of human Survivin (3UIH.pdb) in complex with co-crystallized ligand namely Smac/DIABLO(1–15) peptide (A), and their produced intermolecular interactions (B).

The results of the molecular docking simulations clearly demonstrate that most active ligands were complexed to the targeted receptor encoded in the PDB basis by 3UIH.pdb, with the lowest binding energies being −5.98 and −5.62 kcal/mol, respectively, which explains the molecular stability of both complexes. The highest cytotoxic activity in cancer cells [19] for hydroxyquinoline derivatives were labeled C24 and C25, which was justified by the production of a variety of chemical bonds, such as the hydrogen bonds detected with Pro8, Pro47, and Arg108 amino acid residues (AA-R) linked to A chain, more than one common Pi-Pi T-shaped bond formed with His17 AA-R, and another common Pi-Alkyl bond fixed with AA-R, as presented in Fig. 9. Regarding the predicted molecules, which were designed as novel hydroxyquinoline derivatives with the best biological activity, we noticed that similar intermolecular interactions were produced toward the same targeted receptor (3UIH.pdb) with binding energies closer than before of −5.83 and −4.97 kcal/mol, respectively. Two hydrogen bonds were detected with Pro8 and Pro47 AA-R in the same chain, as well as the same chemical bond of Pi-Pi T-shaped bond type created with His17 AA-R, and the same Pi-Alkyl bond linked to Pro12 AA-R (see Fig. 10). The resulted intermolecular contacts share common interactions as those detected towards the co-crystallized ligand bound to the human surviving receptor (3UIH.pdb), such as Arg108, Lys112, and Ile44 AA-R, as displayed in Fig. 11.

### 3.9 MD simulation

The thermodynamic stability of the macromolecular complexes of human survivin protein with hydroxyquinoline derivatives 24, 25, pred2 and pred6 were evaluated by performing MD simulation for a time-period of 100ns. The drug-receptor complex should be stable enough for a nanoscaler time range for effective execution of the concerned therapeutic effect. Thus, MD simulation for a time-period of 100ns has been implemented each of the considered macromolecular complex by means of Schrodinger's Desmond software version 2022.4 [74]. The macromolecular target's monomer was constituted of 136 amino acids having 1110 heavy atoms out of the total 2196 atoms. The thermodynamic stability for each of the macromolecular complexes were analyzed by considering their structural alterations as well as RMSD for the macromolecular backbone and bound ligand throughout the simulation. The compound-24 complexed with human survivin receptor constitute seven flexible bonds comprising 29 heavy atoms of 54 atoms in total. The human survivin compound 24 conjugate displayed that the bound ligand demonstrated some initial conformational change and was followed by the attainment of stabilized conformation. The RMSD observed for protein's backbone was fluctuating within the range of 4.0 and 7.0 Å, while the complex ligand compound 24 exerts some initial fluctuation in the receptor's cavity with its RMSD of around 30 Å.

The complexed compound 25 constitutes seven flexible bonds comprising thirty-three heavy atoms of 59 atoms in total. The human survivin-compound 25 conjugate has displayed that the bound ligand has shown a very small fluctuations for attaining stabilized conformation. RMSD observed for the macromolecular protein's backbone was fluctuating within 5.0–8.0 Å range, while the complex ligand compound-25 has shown RMSD ranging between 5.0 and 9.0 Å within the macromolecular cavity.

The complexed compound pred2 constitutes nine flexible bonds that comprise 31 heavy atoms and 58 atoms in total. The human survivin-pred2 conjugate displayed that the bound ligand demonstrated some major conformational changes until the initial 20 ns of the simulation time, but afterwards, it achieved stabilized conformation and maintained the same thoroughly. The RMSD observed for the macromolecular backbone was observed to execute a couple of initial conformational changes until the initial 20 ns and was followed by fluctuation within the allowed RMSD value between 9 and 12 Å.

The complexed compound pred6 constitutes ten flexible bonds comprising 32 heavy atoms of 59 atoms in total. The human survivin-pred6 conjugate has displayed that the complex ligand has shown a major conformational change at 50 ns of the simulation time followed by couple of moves to achieve the most favorable conformation at the end phase of the simulation. The RMSD for the macromolecular chain was observed to be fluctuating in the range of 3.0–8.0 Å, while the ligand's RMSD was raised up to 48 Å.

**Fig. 12:**
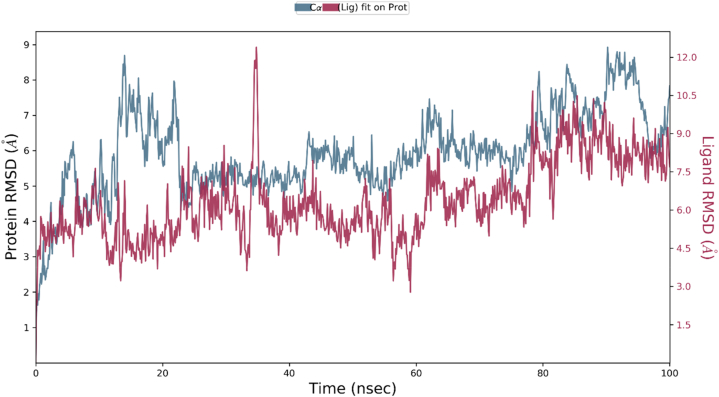
The RMSD for the human survivin Cα chain with bound ligand compound-25 observed during 100ns MD simulation.

The MD simulation have revealed that the bound ligand compound 25 was thermodynamically stable within the macromolecular cavity of the target receptor and its RMSD graph within the macromolecular cavity of human surviving receptor was shown in Fig. 12.

The RMSF value is used to observe the atomic deviation from their initial position within the protein or ligand structure. The RMSF value is an significant criterion to identify their flexibility and dynamic behavior of the macromolecular complex. Protein RMSF directly correlates with the stability as well as dynamic behavior protein. The MD-based evaluation of the thermodynamic stability and structural behavior of the linked human survivin receptor with ligand compound 24 revealed that the RMSF value for the macromolecular Cα chain was fluctuating within 1.5–4.5 Å, while for the complexed ligand compound 24, it was found to be ranging within 12.0–13.0 Å. MD evaluation of human survivin complexed by compound 25 has concluded that the RMSF for Cα backbone was found to be within 1.5–5.0 Å, while for ligand compound 24 it was found to be ranging from 3.0 to 4.0 Å. The RMSF value for human survivin with compound pred2 was found to be within 2.0–6.0 Å for the Cα backbone, while the average variation for the complexed compound pred2 was found to be within 13.0–15.0 Å.

RMSF for human survivin with compound pred6 was found to be within 1.5–4.5 Å for Cα backbone while complexed compound pred6 average variation was found to be within 20.0–21.0 Å.

**Fig. 13:**
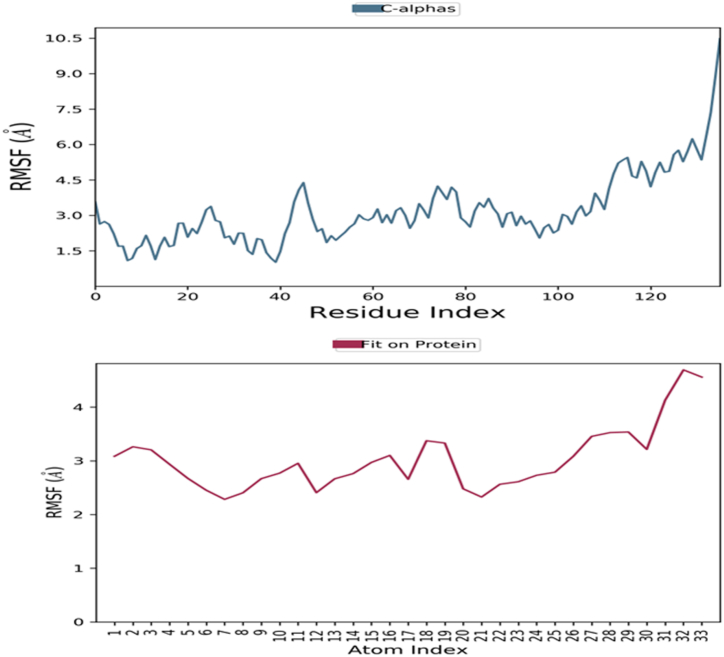
RMSF for survivin-compound 25 complex detected while executing MD simulation.

MD simulation have revealed that the compound 25 was has executed the RMSF value within the acceptable range within the active binding site of the macromolecular cavity of human survivin receptor. Fig. 13 demonstrates the RMSF of the human survivin complexed with compound 25.

The thermodynamic stability of a receptor-ligand complex during an MD simulation is determined by the number and types of chemical interactions existing between the receptor and the bound ligand. Generally hydrophobic contacts, ionic interactions, and hydrogen bonds plays an important role in the drug-receptor interaction. This stability was assessed by consistently tracking the strength and type of these interactions throughout by performing MD modelling of the 3 macromolecular complexes In the course of the simulation, ligand compound 24 interacted with human survivin through the creation a hydrophobic bonds to the amino acids Lys129, Lys130, Val131, Arg133, Ala134, Ile135, and Leu138, whereas Glu126, Arg133, and Gln137 via hydrogen bonds and amino acids Glu123, Glu126, Arg133, Glu136, and Ala139 were found to interact via water bridges. During the simulation, compound 25 was observed to interact with the survivin through the creation of hydrophobic bonds to the relevant amino acids Pro8, Pro12, His17, Lys23, Ile44, Cys46, Phe58, Phe61, and Lys112, whereas Gln11, and Arg108 using hydrogen bonding and amino acid Pro8, Ala9, Gln11, Phe58, and Arg108 interact via water bridges.

**Fig. 14:**
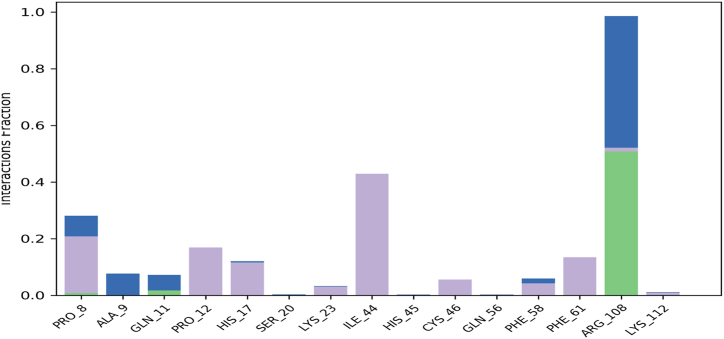
Chemical interactions observed between human survivin and compound 25 observed during 100ns MD simulation involves hydrophobic interactions represented as purple bars, water bridges represented as blue bars, and hydrogen bonds represented as green bars. (For interpretation of the references to color in this figure legend, the reader is referred to the Web version of this article.)

During the simulation, the pred2 ligand was observed interacting with human survivin through the establishment a hydrophobic link to the amino acids Lys23, Ala32, Arg37, Pro47, Pro52, and Pro69, whereas Asn24, Gly30, and Pro47 via hydrogen bonds and the amino acids Glu29, Cys31, Glu36, Glu49Asp70, Asp72, and Lys90 were interaction through water bridges. It was found that the pred6 ligand interacted with the human survivin through the formation to hydrophobic links at the amino acids Pro8, Ala9, Pro12, His17, Lys23, Lue28, Ile44, Cys46, Pro52, Leu54, Phe61, Arg108, Lys112, Phe124, Ala128, Ala134, and Ile135, whereas Pro26, His45, Pro47, and Thr48, via hydrogen bonds and amino acid Thr5, Leu6, Gln11, Glu49, Glu63, Asp105, Glu123, Glu125, Thr127, and Glu136 are linked by hydraulic bridges. MD simulation for 100 ns have revealed that the compound 25 complexed with human surviving receptor is showing the most stabilized interaction throughout the simulation period and Fig. 14 shows the interaction between human survivin and compound 25. The resultant MD simulation output files generated by Desmond software for all the macromolecular complexes considered in the current study were provided as supplementary material.

### 3.10 Design of new compounds

The primary objective of this study is to develop novel hydroxyquinoline scaffold-derived selective survivin inhibitors with insights that were gained from the 2D-QSAR investigations. Nine pyrrolopyrimidine derivatives (Pred1, Pred2, Pred3, Pred4, Pred5, Pred6, Pred7, Pred8, and Pred9) were designed to enhance the inhibitory efficacy against MDA-MB-435, as depicted in Table 5.

**Table 5:** Calculated parameter values fornew molecules and the predicted activity.

Comp	Bend	E_HOMO_	pIC_50__pred
red1	**5.8584**	**−0.19412**	**6.46**
Pred2	**5.6236**	**−0.18347**	**6.68**
Pred3	**6.0125**	**−0.19261**	**6.48**
Pred4	**6.7257**	**−0.19513**	**6.39**
Pred5	**6.4189**	**−0.18703**	**6.57**
Pred6	**5.0599**	**−0.17675**	**6.85**
Pred7	**5.7437**	**−0.19699**	**6.41**
Pred8	**6.5642**	**−0.19957**	**6.31**
Pred9	**5.8921**	**−0.19010**	**6.54**

These additional compounds' descriptors were computed using the same methodology as the molecules in the series under investigation. The RLM model that is presented in Table 6 yielded the anticipated activity of the novel compounds. The table indicates that the novel candidate compounds displayed greater inhibitory activity in comparison with the compounds in the series under consideration.

**Table 6:**
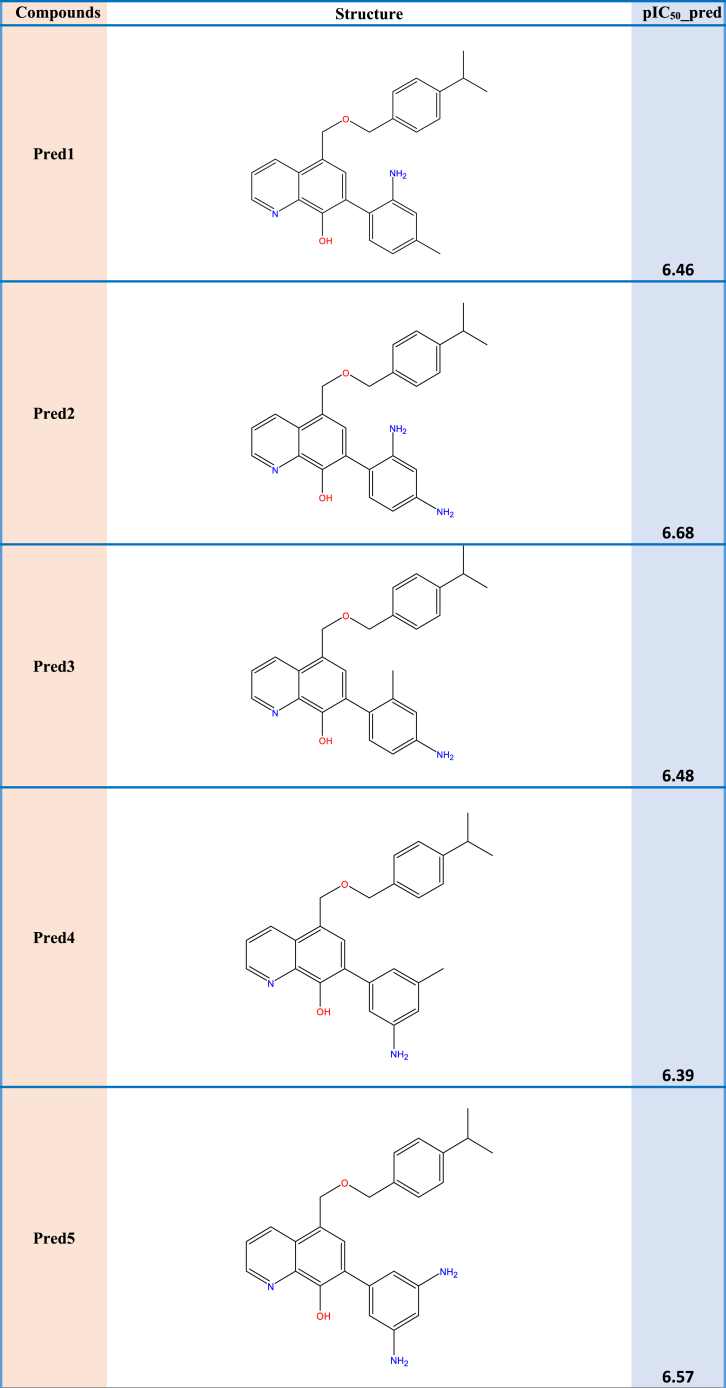

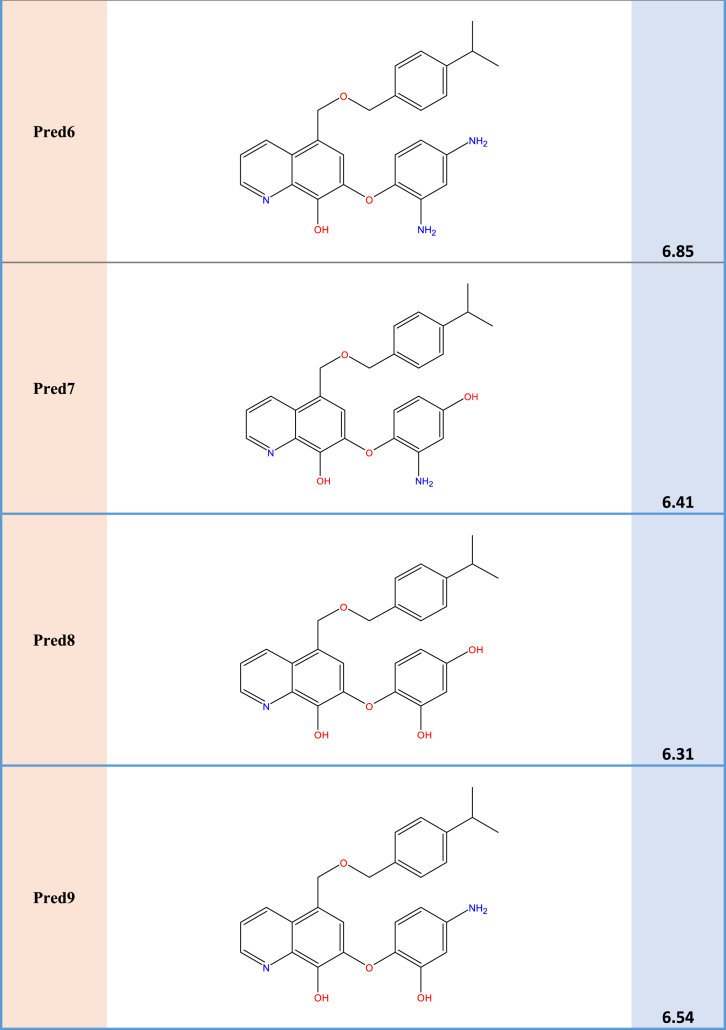
The new compounds' structures and their pIC50 predicted on basis of the 2D-QSAR model.

### 3.11 Lipinski's rule

The newly suggested compounds (Table 7) adhere to Lipinski's Rule of Five, indicating potential for good oral bioavailability. These favorable physicochemical properties make them promising drug candidates. However, it's essential to remember that Lipinski's Rule of Five provides initial guidelines, and further in-depth studies, including preclinical evaluations, are necessary to fully assess their drug development potential.

**Table 7:** Lipinski's rule as applied to the newly designed compounds and their drug-like properties.

	**TPSA**	**n- Rotatable** **Bonds**	**MW**	**LogP**	**n-HBA**	**n-HBD**	**Violations**				**Synthetic accessibility**	**Toxicity**
							Lipinski	Veber	Egan	Muegge		AMES toxicity
**Rule**	<140 A^2^	<10	<500 Da	≤5	<10	<5	≤2	≤2	≤2	≤2	0<*S.A*<10	**Categorical** **(yes/no)**
Pred1	**68.37**	**6**	**412.52**	**3.91**	**3**	**2**	**Yes**	**Yes**	**No**	**No**	**3.30**	**No**
Pred2	**94.39**	**6**	**413.51**	**3.12**	**3**	**3**	**Yes**	**Yes**	**Yes**	**Yes**	**3.31**	**No**
Pred3	**68.37**	**6**	**412.52**	**3.78**	**3**	**2**	**Yes**	**Yes**	**No**	**No**	**3.22**	**No**
Pred4	**68.37**	**6**	**412.52**	**3.91**	**3**	**2**	**Yes**	**Yes**	**No**	**No**	**3.24**	**No**
Pred5	**94.39**	**6**	**413.51**	**3.30**	**3**	**3**	**Yes**	**Yes**	**Yes**	**Yes**	**3.23**	**No**
Pred6	**103.62**	**7**	**429.51**	**3.44**	**4**	**3**	**Yes**	**Yes**	**Yes**	**Yes**	**3.44**	**No**
Pred7	**97.83**	**7**	**430.50**	**3.48**	**5**	**3**	**Yes**	**Yes**	**Yes**	**Yes**	**3.37**	**No**
Pred8	**92.04**	**7**	**431.48**	**3.60**	**6**	**3**	**Yes**	**Yes**	**Yes**	**Yes**	**3.22**	**No**
Pred9	**97.83**	**7**	**430.50**	**3.18**	**5**	**3**	**Yes**	**Yes**	**Yes**	**Yes**	**3.28**	**No**

### 3.12 ADMET properties

All nine predicted compounds are currently being investigated using the pkCSM tool in ADMET in silico studies [75]. The ADMET properties of the nine newly selected compounds have been predicted in silico using the tool. Table 8 presents the computed results of the ADMET prediction.

**Table 8:** ADMET properties.

models	Properties												
	Absorption		Distribution		Metabolism							Excretion	Toxicity
	**Intestinal absorption** **(human)**	**P-Gp** **substrate**	**VDss** **(human)**	**CNS permeability**	**CYP450**							**Total** **clearance**	**AMES toxicity**
					**Substrate**		**Inhibitor**						
					**2D6**	**3A4**	**1A2**	**2C19**	**2C9**	**2D6**	**3A4**		
**Unity**	**Numeric (%absorbed)**	**Categorical** **(yes/no)**	**Numeric** **(Log L** **kg-1)**	**Numeric (Log PS)**	**Categorical (YES/NO)**							**Numeric (log mL min**^−1^**kg**^−1^**)**	**Categorical** **(yes/no)**
**Predicted values**													
Pred1	**90.931**	**Yes**	**−0.444**	**−1.647**	**No**	**Yes**	**No**	**Yes**	**Yes**	**No**	**Yes**	**0.386**	**No**
Pred2	**93.951**	**Yes**	**−0.574**	**−1.952**	**No**	**Yes**	**No**	**Yes**	**Yes**	**No**	**Yes**	**0.401**	**No**
Pred3	**90.931**	**Yes**	**−0.444**	**−1.639**	**No**	**Yes**	**No**	**Yes**	**Yes**	**No**	**Yes**	**0.32**	**No**
Pred4	**90.931**	**Yes**	**−0.444**	**−1.623**	**No**	**Yes**	**No**	**Yes**	**Yes**	**No**	**Yes**	**0.31**	**No**
Pred5	**93.951**	**Yes**	**−0.574**	**−1.932**	**No**	**Yes**	**No**	**Yes**	**Yes**	**No**	**Yes**	**0.325**	**No**
Pred6	**93.744**	**Yes**	**−0.704**	**−2.054**	**No**	**Yes**	**No**	**Yes**	**Yes**	**No**	**Yes**	**0.151**	**No**
Pred7	**94.607**	**Yes**	**−0.774**	**−2.087**	**No**	**Yes**	**No**	**Yes**	**Yes**	**No**	**Yes**	**0.168**	**No**
Pred8	**95.47**	**Yes**	**−0.842**	**−2.969**	**No**	**Yes**	**No**	**Yes**	**Yes**	**No**	**Yes**	**0.155**	**No**
Pred9	**94.607**	**Yes**	**−0.774**	**−2.074**	**No**	**Yes**	**No**	**Yes**	**Yes**	**No**	**Yes**	**0.212**	**No**

Based on the data in Table 8, we can make several conclusions.

- A score of less than 30 % indicates poor intestinal absorption in humans. All the predicted compounds exhibited an absorption value of greater than 90 %, which suggests that they are absorbed well by the human gut.
- One pharmacokinetic parameter that provides insight into drug distribution between blood plasma and tissues is the volume of distribution (VDss). A low VDss indicates that a drug is primarily distributed in plasma, whereas a high VDss suggests significant tissue distribution. The predicted VDss values for these compounds indicate that they are primarily distributed in plasma. The partition coefficient (LogP) is used to measure the distribution of a drug between two immiscible phases, typically blood and tissue. It is often used to assess how a drug is distributed between red blood cells and plasma. A LogP value of less than 5 indicates better distribution. The LogP values of less than 4 for all the predicted compounds confirm that these compounds have positive distribution across blood and tissue. According to the CNS index, compounds with a LogP value exceeding 3 are generally considered to have poor central nervous system (CNS) permeability [76]. Every predicted compound demonstrated enhanced ability to penetrate the central nervous system (CNS).
- Cytochrome P450 (CYP) is a critical detoxification enzyme that is part of the body's metabolism. CYP enzymes are found in every tissue of the body. This enzyme aids in the excretion of foreign compounds by oxidizing them. Cytochrome CYP inhibits a wide range of medications and can also activate some of them. The metabolism of a drug may be affected by inhibitors of this enzyme, which could have the opposite effect. Therefore, determining a compound's ability to inhibit cytochromes (CYPs) is essential. Seventeen CYP categories have been identified in humans to date. Only CYP3A4, CYP1A2, CYP2C9, CYP2C19, and CYP2D6 are responsible for the biotransformation of more than 90 % of drugs that pass the first stage of metabolism, even though only CYP1, CYP2, CYP3, and CYP4 are involved in drug metabolism [77]. CYP2D6 and CYP3A4 are the primary isoforms responsible for drug metabolism. Analysis of the predicted compounds' metabolic properties suggests their potential dual role as both substrates and inhibitors of CYP3A4. This dual nature could influence their pharmacokinetics, potentially affecting their rate of elimination and, consequently, their time spent at the therapeutic target before being metabolized.
- To optimize medication dosing for stable concentrations, it's essential to consider clearance rates, influenced by liver metabolism and kidney excretion [78]. A lower clearance index indicates a longer drug half-life. We evaluated the clearance index of our predicted compounds to assess their persistence in the body. All compounds exhibited a total clearance index below 0.5, suggesting prolonged retention. This prolonged presence likely contributes to their effectiveness at inhibiting survivin, even at very low doses.
- Assessing toxicity is a crucial step in drug development. It is essential to verify the safety of precursor compounds before proceeding. The Ames test is a standard method used to evaluate the mutagenic potential of a compound [79]. Therefore, we assessed the toxicity of each predicted compound using in silico Ames test predictions. No toxicity was predicted for any of the compounds. Additionally, in silico ADMET evaluation revealed that all predicted compounds met the assessed pharmacokinetic criteria.
- Consequently, by inhibiting the enzymatic activity of the survivin protein, the predicted compounds hold potential as future cancer treatments. These compounds can serve as a foundation for developing new molecules with enhanced biological profiles and expanded therapeutic applications.

## 4 Conclusion

This study was conducted to develop new MX-106 hydroxyquinoline drugs as novel inhibitors potent and selective survivin. For this reason, a 2D-QSAR analysis was conducted to investigate the structural factors that influence the biological activity of 31 MX-106 analogues that act as BIRC5 inhibitors. The MLR analysis method that was used to build the QSAR model demonstrated exceptionally strong predictive power, which indicates its reliability in forecasting the biological inhibitory activities of the BIRC5 receptor. An equation analysis of the developed QSAR model revealed two key descriptors (Band and EHOMO) that significantly influence the biological inhibitory activity of the BIRC5 receptor. These findings highlight the importance of these descriptors in designing potent compounds that target the BIRC5 receptor for potential anticancer applications. Based on the results obtained from the MLR equation study, we developed nine new molecules with the most potent BIRC5 inhibitory activity. The subsequent evaluation of the pharmacokinetic properties of these selected molecules revealed that all had acceptable pharmacokinetic characteristics. Additionally, an in silico evaluation of the ADMET features for the newly synthesized molecules indicated that they possessed favorable pharmacokinetic profiles. Thus, these molecules have the potential to be valuable new agents for cancer treatment since they can effectively inhibit the enzymatic activity of the BIRC5 protein. The information presented in this study opens up numerous opportunities for medicinal chemists to develop more potent anticancer drug candidates by using the molecular structure of these compounds to design novel derivatives with enhanced anticancer activities.

In our future research, we plan to develop 3D-QSAR models based on the series of MX-106 hydroxyquinoline derivatives.

## Funding statement

This research was funded by the Researchers Supporting Project (No. RSPD2024R566), King Saud University, Riyadh, Saudi Arabia.

## Data availability statement

Data included in article/supplementary material/referenced in article.

## CRediT authorship contribution statement

**Mourad Aloui:** Writing – original draft, Methodology, Investigation, Data curation, Conceptualization. **Mohamed El fadili:** Investigation, Formal analysis, Conceptualization. **Somdutt Mujwar:** Supervision, Software, Resources. **Sara Er-rahmani:** Writing – review & editing, Resources. **Hatem A. Abuelizz:** Resources, Project administration. **Mohammed Er-rajy:** Methodology, Investigation, Conceptualization. **Sara Zarougui:** Writing – original draft, Formal analysis. **Menana Elhallaoui:** Visualization, Supervision.

## Declaration of competing interest

The authors declare that they have no known competing financial interests or personal relationships that could have appeared to influence the work reported in this paper.

## References

[1] Naishima N.L., Faizan S., Raju R.M., Sruthi A.S.V.L., Ng V., Sharma G.K., Vasanth K.S., Shivaraju V.K., Ramu R., Kumar B.P. (2023). Design, synthesis, analysis, evaluation of cytotoxicity against MCF-7 breast cancer cells, 3D QSAR studies and EGFR, HER2 inhibition studies on novel biginelli 1,4-dihydropyrimidines. J. Mol. Struct..

[2] Girgis A.S., Stawinski J., Ismail N.S.M., Farag H. (2012). Synthesis and QSAR study of novel cytotoxic spiro[3H-indole-3,2′(1′H)-pyrrolo[3,4-c]pyrrole]-2,3′,5′(1H,2′aH,4′H)-triones. Eur. J. Med. Chem..

[3] Ghose A., Viswanadhan V. (2001). Software, Tools, and Applications in Drug Discovery.

[4] Cheung C.H.A., Huang C.-C., Tsai F.-Y., Lee J.Y.-C., Cheng S.M., Chang Y.-C., Huang Y.-C., Chen S.-H., Chang J.-Y. (2013). Survivin – biology and potential as a therapeutic target in oncology. OncoTargets Ther..

[5] Ryan B.M., O'Donovan N., Duffy M.J. (2009). Survivin: a new target for anti-cancer therapy. Cancer Treat Rev..

[6] Altieri D.C. (2010). Survivin and IAP proteins in cell-death mechanisms. Biochem. J..

[7] Martínez-García D., Manero-Rupérez N., Quesada R., Korrodi-Gregório L., Soto-Cerrato V. (2019). Therapeutic strategies involving survivin inhibition in cancer. Med. Res. Rev..

[8] Li D., Hu C., Li H. (2018). Survivin as a novel target protein for reducing the proliferation of cancer cells. Biomed. Rep.

[9] Aloui M., Er-rajy M., Imtara H., Goudzal A., Zarougui S., El fadili M., Arthur D.E., Mothana R.A., Noman O.M., Tarayrah M., Menana E. (2024). QSAR modelling, molecular docking, molecular dynamic and ADMET prediction of pyrrolopyrimidine derivatives as novel Bruton's tyrosine kinase (BTK) inhibitors. Saudi Pharm. J..

[10] Bailly C. (2012). Contemporary challenges in the design of topoisomerase II inhibitors for cancer chemotherapy. Chem. Rev..

[11] Danishuddin, Khan A.U. (2016). Descriptors and their selection methods in QSAR analysis: paradigm for drug design. Drug Discov. Today.

[12] Sun H., Stuckey J.A., Nikolovska-Coleska Z., Qin D., Meagher J.L., Qiu S., Lu J., Yang C.-Y., Saito N.G., Wang S. (2008). Structure-based design, synthesis, evaluation, and crystallographic studies of conformationally constrained smac mimetics as inhibitors of the X-linked inhibitor of apoptosis protein (XIAP). J. Med. Chem..

[13] Zhang B., Li H., Yu K., Jin Z. (2022). Molecular docking-based computational platform for high-throughput virtual screening. CCF Trans. High Perform. Comput..

[14] Amin SkA., Kumar J., Khatun S., Das S., Qureshi I.A., Jha T., Gayen S. (2022). Binary quantitative activity-activity relationship (QAAR) studies to explore selective HDAC8 inhibitors: in light of mathematical models, DFT-based calculation and molecular dynamic simulation studies. J. Mol. Struct..

[15] Yamanaka K., Nakata M., Kaneko N., Fushiki H., Kita A., Nakahara T., Koutoku H., Sasamata M. (2011). YM155, a selective survivin suppressant, inhibits tumor spread and prolongs survival in a spontaneous metastatic model of human triple negative breast cancer. Int. J. Oncol..

[16] Roy K., Singh N., Kanwar R.K., Kanwar J.R. (2016). Survivin modulators: an updated patent review (2011 - 2015). Recent Patents Anticancer Drug Discov.

[17] Iwai M., Minematsu T., Li Q., Iwatsubo T., Usui T. (2011). Utility of P-glycoprotein and organic cation transporter 1 double-transfected LLC-PK1 cells for studying the interaction of YM155 monobromide, novel small-molecule survivin suppressant, with P-glycoprotein. Drug Metab. Dispos..

[18] Xiao M., Wang J., Lin Z., Lu Y., Li Z., White S.W., Miller D.D., Li W. (2015). Design, synthesis and structure-activity relationship studies of novel survivin inhibitors with potent anti-proliferative properties. PLoS One.

[19] Albadari N., Deng S., Chen H., Zhao G., Yue J., Zhang S., Miller D.D., Wu Z., Li W. (2021). Synthesis and biological evaluation of selective survivin inhibitors derived from the MX-106 hydroxyquinoline scaffold. Eur. J. Med. Chem..

[20] Österberg T., Norinder U. (2001). Prediction of drug transport processes using simple parameters and PLS statistics the use of ACD/logP and ACD/ChemSketch descriptors. Eur. J. Pharm. Sci..

[21] Milne G.W.A. (2010). Software review of ChemBioDraw 12.0. J. Chem. Inf. Model..

[22] Parr R.G., Yang W. (1995). Density-functional theory of the electronic structure of molecules. Annu. Rev. Phys. Chem..

[23] Zhang Y., Xu X., Goddard W.A. (2009). Doubly hybrid density functional for accurate descriptions of nonbond interactions, thermochemistry, and thermochemical kinetics. Proc. Natl. Acad. Sci..

[24] Gaussian 09 Citation | Gaussian com,(n.d.). https://gaussian.com/g09citation/(accessed September 22, 2024).

[25] Gupta M.K., Gupta S., Rawal R.K., Puri M., Pathak Y., Sutariya V.K., Tipparaju S., Moreno W. (2016). Artif. Neural Netw. Drug Des. Deliv. Dispos..

[26] Roy K., Mitra I. (2011). On various metrics used for validation of predictive QSAR models with applications in virtual screening and focused library design. Comb. Chem. High Throughput Screen..

[27] Er-rajy M., El fadili M., Mrabti N.N., Zarougui S., Elhallaoui M. (2022). QSAR, molecular docking, ADMET properties in silico studies for a series of 7-propanamide benzoxaboroles as potent anti-cancer agents. Chin. J. Anal. Chem..

[28] Salt D.W., Yildiz N., Livingstone D.J., Tinsley C.J. (1992). The use of artificial neural networks in QSAR. Pestic. Sci..

[29] Kůrková V. (1992). Kolmogorov's theorem and multilayer neural networks. Neural Netw.

[30] Golbraikh A., Tropsha A. (2002). Beware of q2. J. Mol. Graph. Model..

[31] Rücker C., Rücker G., Meringer M. (2007). y-Randomization and its Variants in QSPR/QSAR. J. Chem. Inf. Model..

[32] Roy K., Mitra I. (2011). On various metrics used for validation of predictive QSAR models with applications in virtual screening and focused library design. Comb. Chem. High Throughput Screen..

[33] Daoui O., Elkhattabi S., Chtita S., Elkhalabi R., Zgou H., Benjelloun A.T. (2021). QSAR, molecular docking and ADMET properties in silico studies of novel 4,5,6,7-tetrahydrobenzo[D]-thiazol-2-Yl derivatives derived from dimedone as potent anti-tumor agents through inhibition of C-Met receptor tyrosine kinase. Heliyon.

[34] Hadni H., Elhallaoui M. (2020). 3D-QSAR, docking and ADMET properties of aurone analogues as antimalarial agents. Heliyon.

[35] Tropsha A., Gramatica P., Gombar V.K. (2003). The importance of being earnest: validation is the absolute essential for successful application and interpretation of QSPR models, QSAR comb. Sci..

[36] Kuruvilla T.K., Prasana J.C., Muthu S., George J., Mathew S.A. (2018). Quantum mechanical and spectroscopic (FT-IR, FT-Raman) study, NBO analysis, HOMO-LUMO, first order hyperpolarizability and molecular docking study of methyl[(3R)-3-(2-methylphenoxy)-3-phenylpropyl]amine by density functional method. Spectrochim. Acta. A. Mol. Biomol. Spectrosc..

[37] Li L., Wu C., Wang Z., Zhao L., Li Z., Sun C., Sun T. (2015). Density functional theory (DFT) and natural bond orbital (NBO) study of vibrational spectra and intramolecular hydrogen bond interaction of l-ornithine–l-aspartate. Spectrochim. Acta. A. Mol. Biomol. Spectrosc..

[38] Subhapriya G., Kalyanaraman S., Surumbarkuzhali N., Vijayalakshmi S., Krishnakumar V. (2015). Investigation of intermolecular hydrogen bonding in 2,3,4,5,6 pentafluorobenzoic acid through molecular structure and vibrational analysis – a DFT approach. J. Mol. Struct..

[39] Zoete V., Cuendet M.A., Grosdidier A., Michielin O. (2011). SwissParam: a fast force field generation tool for small organic molecules. J. Comput. Chem..

[40] Parrinello M., Rahman A. (1981). Polymorphic transitions in single crystals: a new molecular dynamics method. J. Appl. Phys..

[41] El fadili M., Er-rajy M., Ali Eltayb W., Kara M., Assouguem A., Saleh A., Al Kamaly O., Zarougui S., Elhallaoui M. (2023). In-silico screening based on molecular simulations of 3,4-disubstituted pyrrolidine sulfonamides as selective and competitive GlyT1 inhibitors. Arab. J. Chem..

[42] Rani D.U., Begum S., Nithya S., Fadili M.E.L. (2023). Investigation of linagliptin-human serum albumin complex formation using spectroscopic analysis and molecular docking. Oriental Journal of Chemistry | EBSCOhost.

[43] Ajala A., Eltayb W.A., Abatyough T.M., Ejeh S., El fadili M., Otaru H.A., Edache E.I., Abdulganiyyu A.I., Areguamen O.I., Patil S.M., Ramu R. (2023). *In-silico* screening and ADMET evaluation of therapeutic MAO-B inhibitors against Parkinson disease, intell. Pharm. Times.

[44] El fadili M., Er-Rajy M., Kara M., Assouguem A., Belhassan A., Alotaibi A., Mrabti N.N., Fidan H., Ullah R., Ercisli S., Zarougui S., Elhallaoui M. (2022). QSAR, ADMET in silico pharmacokinetics, molecular docking and molecular dynamics studies of novel bicyclo (aryl methyl) benzamides as potent GlyT1 inhibitors for the treatment of schizophrenia. Pharmaceuticals.

[45] El fadili M., Er-rajy M., Imtara H., Kara M., Zarougui S., Altwaijry N., Al kamaly O.M., Al Sfouk A., Elhallaoui M. (2022). 3D-QSAR, ADME-tox in silico prediction and molecular docking studies for modeling the analgesic activity against neuropathic pain of novel NR2B-selective NMDA receptor antagonists. Processes.

[46] El fadili M., Er-rajy M., Abdalla M., Abuelizz H.A., Zarougui S., Alkhulaifi F.M., Alahmady N.F., Shami A., Elhalaoui M. (2023). In-silico investigations of novel tacrine derivatives potency against Alzheimer's disease. Sci. Afr..

[47] (2023). Discovery Studio.

[48] Abechi S.E., Michael A.T., Abduljelil A., Stephen E., Asipita O.H., El fadili M. (2024). Virtual screening and pharmacokinetics analysis of inhibitors against tuberculosis: structure and ligand-based approach. Sci. Afr..

[49] Assaggaf H., El Hachlafi N., El fadili M., Elbouzidi A., Ouassou H., Jeddi M., Alnasser S.M., Qasem A., Attar A., AL-Farga A., Alghamdi O.A., Mehana E.E., Mrabti H.N. (2023). GC/MS profiling, in vitro antidiabetic efficacy of origanum compactum benth. Essential oil and in silico molecular docking of its major bioactive compounds. Catalysts.

[50] Daoui O., Mazoir N., Bakhouch M., Salah M., Benharref A., Gonzalez-Coloma A., Elkhattabi S., Yazidi M.E., Chtita S. (2022). 3D-QSAR, ADME-Tox, and molecular docking of semisynthetic triterpene derivatives as antibacterial and insecticide agents. Struct. Chem..

[51] Benkhaira N., El Hachlafi N., El fadili M., Jeddi M., Abdnim R., Bnouham M., Ibnsouda Koraichi S., Fikri-Benbrahim K. (2023). Unveiling the phytochemical profile, *in vitro* bioactivities evaluation, *in silico* molecular docking and ADMET study of essential oil from *Clinopodium nepeta* grown in Middle Atlas of Morocco. Biocatal. Agric. Biotechnol..

[52] Jeddi M., El Hachlafi N., El Fadili M., Benkhaira N., Al-Mijalli S.H., Kandsi F., Abdallah E.M., Ouaritini Z.B., Bouyahya A., Lee L.-H., Zengin G., Mrabti H.N., Fikri-Benbrahim K. (2023). Antimicrobial, antioxidant, *α*-amylase and *α*-glucosidase inhibitory activities of a chemically characterized essential oil from *Lavandula angustifolia* Mill.,: *in vitro* and *in silico* investigations. Biochem. Syst. Ecol..

[53] Bowers K.J., Chow E., Xu H., Dror R.O., Eastwood M.P., Gregersen B.A., Klepeis J.L., Kolossvary I., Moraes M.A., Sacerdoti F.D., Salmon J.K., Shan Y., Shaw D.E. (2006). Proc. 2006 ACMIEEE Conf. Supercomput..

[54] Bowers K.J., Chow E., Xu H., Dror R.O., Eastwood M.P., Gregersen B.A., Klepeis J.L., Kolossvary I., Moraes M.A., Sacerdoti F.D., Salmon J.K., Shan Y., Shaw D.E. (2006). Proc. 2006 ACMIEEE Conf. Supercomput..

[55] Li Q., Zhang H., Guan S., Du J., Zhang Y., Wang S. (2023). Molecular dynamics simulation of the inhibition mechanism of factor XIa by Milvexian-like macrocyclic inhibitors. Comput. Theor. Chem..

[56] Sharma V., Kumar V., Kumar P. (2013). Heterocyclic chalcone analogues as potential anticancer agents. Anti-Cancer Agents Med. Chem.- Anti-Cancer Agents.

[57] Uddin MdS., Rahman MdA., Kabir MdT., Behl T., Mathew B., Perveen A., Barreto G.E., Bin-Jumah M.N., Abdel-Daim M.M., Ashraf G.M. (2020). Multifarious roles of mTOR signaling in cognitive aging and cerebrovascular dysfunction of Alzheimer's disease. IUBMB Life.

[58] Kumar V., Parate S., Danishuddin, Zeb A., Singh P., Lee G., Jung T.S., Lee K.W., Ha M.W. (2022). 3D-QSAR-Based pharmacophore modeling, virtual screening, and molecular dynamics simulations for the identification of spleen tyrosine kinase inhibitors. Front. Cell. Infect. Microbiol..

[59] Mujwar S. (2021). Computational repurposing of tamibarotene against triple mutant variant of SARS-CoV-2. Comput. Biol. Med..

[60] Mujwar S., Pardasani K. (2023). Molecular docking simulation-based pharmacophore modeling to design translation inhibitors targeting c-di-GMP riboswitch of Vibrio cholera. Lett. Drug Des. Discov..

[61] Mujwar S., Tripathi A. (2022). Repurposing benzbromarone as antifolate to develop novel antifungal therapy for Candida albicans. J. Mol. Model..

[62] Pradhan P., Soni N.K., Chaudhary L., Mujwar S., Pardasani K.R. (2015). In-silico prediction of riboswitches and design of their potent inhibitors for H1N1, H2N2 and H3N2 strains of influenza virus. Biosci. Biotechnol. Res. Asia.

[63] Agrawal N., Mujwar S., Goyal A., Gupta J.K. (2022). Phytoestrogens as potential antiandrogenic agents against prostate cancer: an in silico analysis. Lett. Drug Des. Discov..

[64] Mujwar S., Shah K., Gupta J.K., Gour A. (2021). Docking based screening of curcumin derivatives: a novel approach in the inhibition of tubercular DHFR. Int. J. Comput. Biol. Drug Des..

[65] Shah K., Mujwar S., Krishna G., Gupta J.K. (2020). Computational design and biological depiction of novel naproxen derivative. Assay Drug Dev. Technol..

[66] Er-rajy M., El fadili M., Mujwar S., Zarougui S., Elhallaoui M. (2023). Design of novel anti-cancer drugs targeting TRKs inhibitors based 3D QSAR, molecular docking and molecular dynamics simulation. J. Biomol. Struct. Dyn..

[67] Er-rajy M., Fadili M.E., Mujwar S., Lenda F.Z., Zarougui S., Elhallaoui M. (2023). QSAR, molecular docking, and molecular dynamics simulation–based design of novel anti-cancer drugs targeting thioredoxin reductase enzyme. Struct. Chem..

[68] Kaur A., Mujwar S., Adlakha N. (2016). In-silico analysis of riboswitch of Nocardia farcinica for design of its inhibitors and pharmacophores. Int. J. Comput. Biol. Drug Des..

[69] Shinu P., Sharma M., Gupta G.L., Mujwar S., Kandeel M., Kumar M., Nair A.B., Goyal M., Singh P., Attimarad M., Venugopala K.N., Nagaraja S., Telsang M., Aldhubiab B.E., Morsy M.A. (2022). Computational design, synthesis, and pharmacological evaluation of naproxen-guaiacol chimera for gastro-sparing anti-inflammatory response by selective COX2 inhibition. Molecules.

[70] Alqahtani S. (2017). In silico ADME-Tox modeling: progress and prospects. Expert Opin. Drug Metab. Toxicol..

[71] Hansch C., Leo A., Mekapati S.B., Kurup A. (2004). QSAR and ADME. Bioorg. Med. Chem..

[72] Jin Z., Wang Y., Yu X.-F., Tan Q.-Q., Liang S.-S., Li T., Zhang H., Shaw P.-C., Wang J., Hu C. (2020). Structure-based virtual screening of influenza virus RNA polymerase inhibitors from natural compounds: molecular dynamics simulation and MM-GBSA calculation. Comput. Biol. Chem..

[73] Kiralj R., Ferreira M.M.C. (2009). Basic validation procedures for regression models in QSAR and QSPR studies: theory and application. J. Braz. Chem. Soc..

[74] Desmond | Schrödinger Life Science, (n.d.) https://www.schrodinger.com/platform/products/desmond/(accessed September 22, 2024).

[75] Pires D.E.V., Blundell T.L., Ascher D.B. (2015). pkCSM: predicting small-molecule pharmacokinetic and toxicity properties using graph-based signatures. J. Med. Chem..

[76] El fadili M., Er-rajy M., Ali Eltayb W., Kara M., Imtara H., Zarougui S., Al-Hoshani N., Hamadi A., Elhallaoui M. (2023). An in-silico investigation based on molecular simulations of novel and potential brain-penetrant GluN2B NMDA receptor antagonists as anti-stroke therapeutic agents. J. Biomol. Struct. Dyn..

[77] Šrejber M., Navrátilová V., Paloncýová M., Bazgier V., Berka K., Anzenbacher P., Otyepka M. (2018). Membrane-attached mammalian cytochromes P450: an overview of the membrane's effects on structure, drug binding, and interactions with redox partners. J. Inorg. Biochem..

[78] Benkhaira N., El Hachlafi N., El fadili M., Jeddi M., Abdnim R., Bnouham M., Ibnsouda Koraichi S., Fikri-Benbrahim K. (2023). Dévoilement du profil phytochimique, l’évaluation *in vitro* des bioactivités*,* l’amarrage moléculaire *silico* et l’étude ADMET de l’huile essentielle de *Clinopodium nepeta* cultivée au Moyen Atlas du Maroc. Biocatal. Agric. Biotechnol..

[79] Shinu P., Sharma M., Gupta G.L., Mujwar S., Kandeel M., Kumar M., Nair A.B., Goyal M., Singh P., Attimarad M., Venugopala K.N., Nagaraja S., Telsang M., Aldhubiab B.E., Morsy M.A. (2022). Computational design, synthesis, and pharmacological evaluation of naproxen-guaiacol chimera for gastro-sparing anti-inflammatory response by selective COX2 inhibition. Molecules.

